# *Candida albicans*-induced ubiquitination of EGFR reveals novel host–fungal interaction pathways

**DOI:** 10.1128/mbio.03448-25

**Published:** 2026-01-12

**Authors:** Léa Lortal, James S. Griffiths, Emily L. Priest, Alexander Kempf, Olivia K. A. Paulin, Nicole O. Ponde, Antzela Tsavou, Don N. Wickramasinghe, Andrew Donkin, Claire M. Lyon, Olivia W. Hepworth, Jonathan P. Richardson, Julian R. Naglik

**Affiliations:** 1 Centre for Host-Microbiome Interactions, Faculty of Dentistry, Oral and Craniofacial Sciences, King’s College London 61139 , London, United Kingdom; 2 Department of Biochemistry and Biophysics, University of California San Francisco 117253 , San Francisco, California, USA; 3 Department of Molecular Genetics, University of Toronto, Toronto, Canada; 4 Division of Rheumatology and Clinical Immunology, University of Pittsburgh 558632, Pittsburgh, Pennsylvania, USA; 5 Department of Medicine, Division of Infectious Diseases, Columbia University, New York, New York, USA; Hebrew University of Jerusalem Robert H Smith Faculty of Agriculture Food and Environment, Rehovot, Israel

**Keywords:** *Candida albicans*, candidalysin, epidermal growth factor receptor, oropharyngeal candidiasis, ubiquitin

## Abstract

*Candida albicans* causes severe mucosal and systemic infections, with hypha formation playing a key role in its virulence. Hyphal invasion via endocytosis is mediated predominantly through interactions between Als3p and the epidermal growth factor receptor (EGFR). Subsequent EGFR activation by candidalysin, a hyphal-secreted cytolytic peptide toxin encoded by the *ECE1* gene, induces receptor signaling and immune responses. While EGFR ubiquitination critically regulates receptor trafficking and signaling, its involvement during *C. albicans* infection has remained unexplored. Here, we demonstrate that *C. albicans* induces EGFR ubiquitination, leading to altered trafficking and lysosomal degradation in an *ECE1*- and *ALS3*-dependent manner. This correlates with changes in EGFR ligand expression, adaptor recruitment, and protein ubiquitination in oral epithelial cells. In a mouse model of oropharyngeal candidiasis, wild-type *C. albicans* and *ece1*Δ/Δ and *als3*Δ/Δ mutant strains were found to differentially regulate *Egfr* expression, ubiquitin pathway-associated genes, and protein ubiquitination. Furthermore, conditional EGFR knockout was protective during infection. Together, our findings reveal that *C. albicans* infection modulates the host ubiquitin system, including direct effects on EGFR, highlighting a novel aspect of host–fungal interactions.

**IMPORTANCE:**

*Candida albicans* is a common fungal pathogen that causes both mucosal infections, such as thrush, and life-threatening systemic diseases. A key step in infection is the fungus invading epithelial tissues and activating the host epidermal growth factor receptor (EGFR). We discovered that *C. albicans* alters how EGFR is regulated by inducing its ubiquitination, a modification that leads to receptor degradation. This process depends on two major fungal virulence factors: the adhesin Als3p and Ece1p, the polypeptide that contains the candidalysin toxin. The fungus also broadly increases protein ubiquitination in oral epithelial cells. In a mouse model of oral infection, loss of EGFR in epithelial tissues reduced disease severity, suggesting that the receptor helps the fungus establish infection. These findings reveal a previously unrecognized strategy by which *C. albicans* manipulates protein ubiquitination and regulation in epithelial cells, offering new insights into fungal pathogenesis and potential therapeutic approaches that target host pathways.

## INTRODUCTION

With over a billion individuals impacted globally and an estimated 3.8 million deaths each year, fungal infections represent a critical and often underrecognized global health concern (1–3). *Candida albicans* is a major contributor to this health burden, causing distressing mucosal infections, such as vulvovaginal and oral candidiasis, and life-threatening systemic candidemia (1, 3, 4).

*C. albicans* is a polymorphic fungus that transitions between yeast, pseudo-hyphal, and hyphal forms (5). This morphological switching is a key virulence attribute, with hyphal growth enabling tissue penetration and epithelial invasion (6, 7). Adhesion to epithelial cells is also an essential virulence attribute (7, 8). *C. albicans* hyphae express numerous adhesins, including agglutinin-like sequence 3 (Als3p), hyphal wall protein 1 (Hwp1p), and hyphally regulated cell wall protein 1 (Hyr1p). Als3p mediates adhesion to epithelial receptors such as E-cadherin, while Hwp1p becomes covalently linked to epithelial cells following processing by host transglutaminases (9–12). Once adhered, hyphae invade epithelial cells via two distinct but complementary mechanisms: active penetration (AP) and receptor-induced endocytosis (RIE) (11, 13, 14). In AP, extending hyphae exert mechanical force on epithelial cells and secrete hydrolytic enzymes (14). In contrast, RIE occurs when fungal invasins Als3p and Ssa1p engage host receptors epidermal growth factor receptor (EGFR) and human epidermal growth factor receptor 2 (HER2), thereby inducing endocytosis (15, 16). Additionally, Als3p and Hyr1p can activate E-cadherin, EGFR/HER2, and c-Met multiprotein complexes (17). Both AP and RIE lead to the formation of an invasion pocket, where the invading hyphal tip becomes enveloped by the invaginated host membrane (18).

Within the invasion pocket, *C. albicans* hyphae secrete candidalysin, a cytolytic peptide toxin encoded by the *ECE1* gene (18–23). Ece1p is a polypeptide of 271 amino acids that is sequentially processed by Kex2p and Kex1p to produce eight peptides. Candidalysin is the third peptide (positions 62–92) and is 31 amino acids in length. The toxin is critical for infection, disrupting host cell membranes and activating epithelial immune responses (22, 24–30). Candidalysin also drives EGFR activation through both ligand-dependent and independent mechanisms. Ligand-dependent activation of EGFR results from candidalysin-induced calcium influx into epithelial cells. This activates matrix metalloproteinases, which in turn cleave the surface-tethered ligands epigen, epiregulin, and amphiregulin. Once released from the epithelial cell surface, these ligands bind and activate EGFR (25). In parallel, candidalysin stimulates the p38 signaling pathway, which activates EGFR in a ligand-independent manner (28). EGFR activation leads to downstream extracellular signal-regulated kinase 1/2 (ERK1/2) signaling and cytokine production, promoting neutrophil recruitment and type 17 immunity, both critical for host protection against oral candidiasis (22, 24, 25, 28, 31). Thus, EGFR plays a dual role during infection: it facilitates fungal endocytosis while simultaneously orchestrating protective immune responses.

Typically, EGFR is rapidly internalized into the cell upon activation and trafficked through endosomal compartments, where it is either recycled back to the plasma membrane or targeted for lysosomal degradation (32–35). This trafficking is tightly regulated, as the balance between recycling and degradation determines the intensity and duration of EGFR signaling (36). Ubiquitination of EGFR plays a central role in this process, modulating receptor internalization and trafficking (37–42).

Beyond EGFR regulation, the ubiquitin system is integral to numerous cellular processes, including protein degradation, DNA repair, signal transduction, and immune defense (43–46). Disruptions in ubiquitin dynamics are linked to cancer, neurodegenerative diseases, and immune dysfunction (43, 47–49). Importantly, ubiquitination is a critical process during microbial infection (50, 51), and pathogens can target the ubiquitin system to facilitate infection (52–54).

While *C. albicans* and candidalysin are known to activate EGFR, it remains unclear whether activation involves EGFR ubiquitination and altered receptor trafficking or if *C. albicans* modulates the host ubiquitin system. In this study, we demonstrate that *C. albicans* and candidalysin selectively induce the upregulation of EGFR ligands, ubiquitin pathway-associated genes, and protein ubiquitination in oral epithelial cells. We show that EGFR undergoes ubiquitination in an *ECE1*-driven manner and is targeted for lysosomal degradation at later stages of infection, in a process requiring both *ECE1* and *ALS3*. We also identify the recruitment of key EGFR adaptor proteins Grb2, AP2M1, and HRS during infection. In a mouse model of oropharyngeal candidiasis (OPC), wild-type (WT) *C. albicans* and *ece1*Δ/Δ and *als3*Δ/Δ mutant strains were found to differentially and dynamically regulate *Egfr* expression, ubiquitin pathway-associated genes, and protein ubiquitination. Notably, conditional knockout (cKO) of EGFR in the oral cavity (keratin 14+ [K14] tissues) was protective during OPC, suggesting EGFR plays a crucial role during the establishment of infection. Together, our findings reveal that *C. albicans* infection modulates the host ubiquitin system, including direct effects on EGFR, highlighting a novel aspect of host–fungal interactions.

## RESULTS

### *C. albicans* and candidalysin selectively upregulate EGFR ligands while maintaining stable EGFR expression

**Fig 1:**
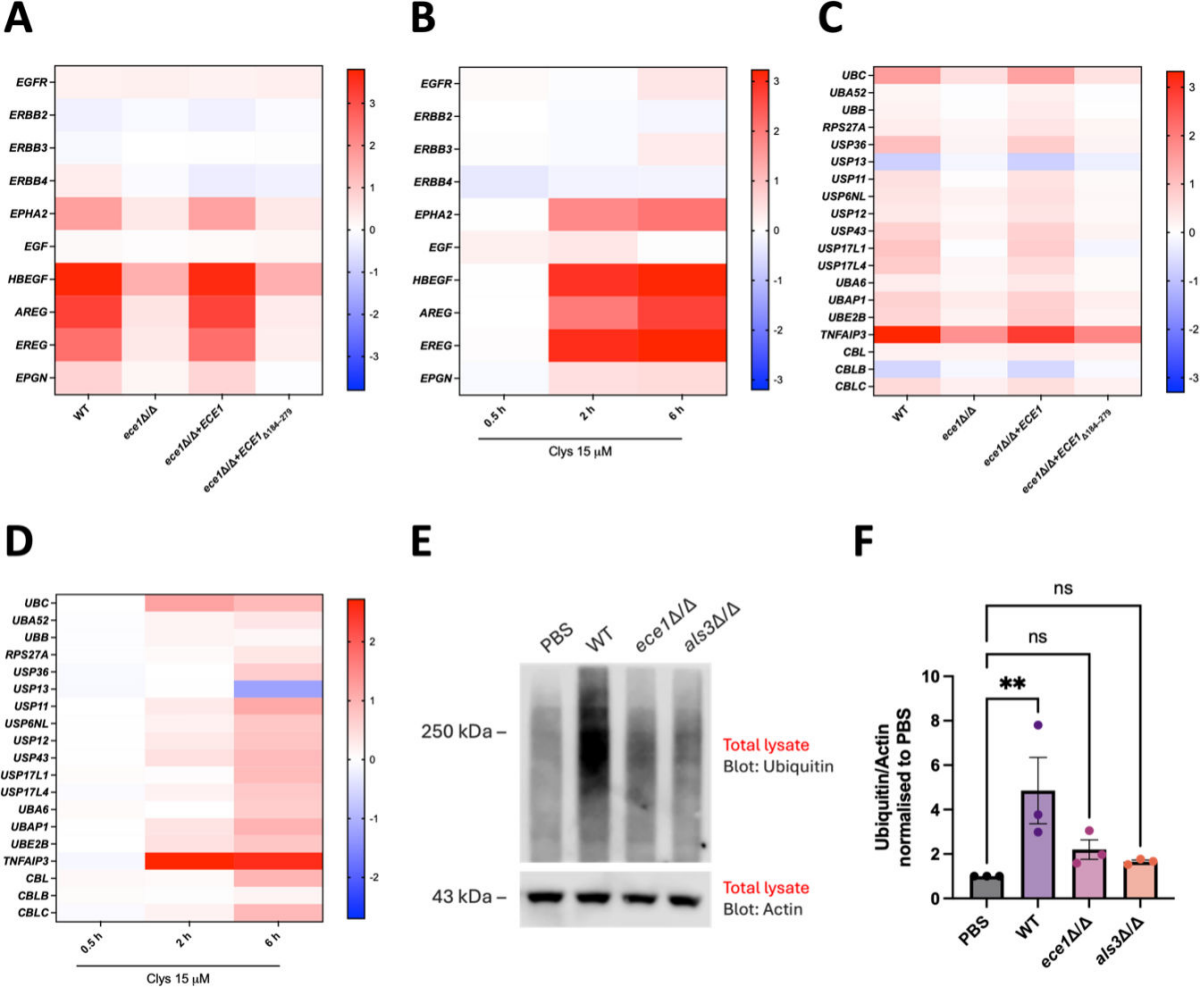
*C. albicans* selectively induces the expression of EGFR ligands and ubiquitin pathway-associated genes and induces protein ubiquitination. (**A–D**) RNA-sequencing heatmap of selected EGFR- and ubiquitin-associated genes comparing the log_2_ fold changes of the indicated conditions relative to the vehicle control (PBS or water). TR146 cells were infected with wild-type (WT) *C. albicans* (BWP17, parental strain), an *ECE1* deletion strain (*ece1*Δ/Δ), an *ECE1* re-integrant strain (*ece1*Δ/Δ + *ECE1*), and a candidalysin deletion strain (*ece1*Δ/Δ + *ECE1*_Δ184–279_) for 4 h (multiplicity of infection [MOI] 10) or treated with an intermediate lytic concentration (15 μM) of candidalysin for 0.5, 2.0, and 6.0 h. RNA was extracted, and samples were sent for RNA sequencing. Data are representative of three independent experiments. (**E**) Western blot of oral epithelial cell lysate for ubiquitin. TR146 cells were infected with WT *C. albicans* (SC5314) and *ece1*Δ/Δ and *als3*Δ/Δ strains at an MOI of 5 or stimulated with PBS (vehicle control) for 6 h. Whole-cell lysates (10 µg) were electrophoresed on gradient gels and analyzed by Western blot to detect the presence of ubiquitin. α-Actin was used as a loading control. Data are representative of three independent experiments. (**F**) Densitometry analysis of panel **E**; graphs represent the average of three independent experiments (±SEM); individual points represent independent experiments. All values are normalized to the vehicle control (PBS), which is set as 1. Statistical significance was assessed using the Kruskal–Wallis test with Dunn’s multiple comparison test. **P < 0.01. ns, not significant.

To investigate EGFR trafficking, we first assessed the expression of *EGFR*, key EGFR co-receptors, and EGFR ligands in response to *C. albicans* infection or stimulation with candidalysin. TR146 oral epithelial cells were infected with WT *C. albicans* (BWP17, parental strain; derivative of SC5314), an *ECE1* deletion strain (*ece1*Δ/Δ), an *ECE1* re-integrant strain (*ece1*Δ/Δ + *ECE1*), and a candidalysin-deficient strain (*ece1*Δ/Δ + *ECE1*_Δ184–279_) for 4 h or treated with candidalysin for 0.5, 2.0, or 6.0 h. RNA sequencing analysis revealed that *C. albicans* infection did not induce significant changes in *EGFR* gene expression compared to vehicle-treated cells (Fig. 1A). Similarly, candidalysin treatment did not affect *EGFR* expression at 2 h but induced a minor increase in *EGFR* gene expression at 6 h (1.32-fold change, adjusted *P* value [*padj*] < 0.001) (Fig. 1B). The other members of the EGFR family (ErbB2/HER2, ErbB3/HER3, and ErbB4/HER4), which are not activated during *C. albicans* infection (25), showed no upregulation (Fig. 1A). However, *EPHA2*, which forms a complex with EGFR and is also activated during *C. albicans* infection (55), was significantly upregulated in a candidalysin-dependent manner (4.2-fold change, *padj* < 0.001) (Fig. 1A and B).

EGFR is activated by many ligands, including epidermal growth factor (EGF), heparin-binding EGF (HB-EGF), transforming growth factor-alpha (TGF-α), amphiregulin (AREG), betacellulin (BTC), epiregulin (EREG) and epigen (EPGN) (56), and receptor fate can vary depending on the specific ligand and its concentration. While HB-EGF and BTC are associated with EGFR degradation, TGF-α, EREG, AREG, and EPG are linked to EGFR recycling (32–34, 57). In addition, low concentrations of EGF (≤1 ng/mL) are linked to EGFR recycling, whereas high concentrations (≥10 ng/mL) are linked to degradation (58), demonstrating that the fate of EGFR also depends on ligand concentration.

We previously quantified the EGFR ligands released from the epithelial surface following candidalysin treatment and found that AREG, EREG, and EPGN were predominantly released (25). In the RNA-sequencing data, expression of *AREG* was significantly upregulated in WT-infected cells (9.4-fold change, *padj* < 0.001), which required *ECE1*, as *AREG* expression was reduced in strains lacking *ECE1* (*ece1*Δ/Δ) or the candidalysin-encoding region (*ece1*Δ/Δ + *ECE1*_Δ184–279_). Re-introduction of *ECE1* in the *ece1*Δ/Δ strain restored *AREG* expression (Fig. 1A). Consistently, candidalysin treatment significantly increased *AREG* expression in TR146 cells compared to control cells at both 2 and 6 h (Fig. 1B). Gene expression of *EREG* and *EPGN* followed similar trends, with significant increases observed in response to both WT *C. albicans* (5.8- and 1.8-fold, *padj* < 0.001 and <0.05, respectively) and candidalysin (9.4- and 1.5-fold, *padj* < 0.001 and <0.01, respectively) (Fig. 1A and B). In contrast, the expression of *EGF*, which is not released during infection (25), remained stable under both conditions. Notably, while HB-EGF release was not detected previously (25), the RNA-sequencing data showed that *HBEGF* was one of the most upregulated genes in response to WT *C. albicans* (13.3-fold change, *padj* < 0.001) and candidalysin treatment (9.0-fold change at 6 h, *padj* < 0.001). These data demonstrate that epithelial responses to *C. albicans* and candidalysin involve the selective upregulation of EGFR ligands while maintaining stable EGFR expression, implying a targeted modulation of host signaling.

### *C. albicans* induces ubiquitin pathway-associated genes and protein ubiquitination in oral epithelial cells

Ubiquitination is essential for EGFR degradation, signaling regulation, and broader cellular processes, including immune responses and protein homeostasis (37, 43, 59). To explore the role of ubiquitination in the epithelial response to *C. albicans* infection and candidalysin, we examined the expression of ubiquitin and genes in the ubiquitin pathway.

In humans, ubiquitin is encoded by four genes: Ubiquitin B (*UBB*), Ubiquitin C (*UBC*), Ubiquitin A-52 Residue Ribosomal Protein Fusion Product 1 (*UBA52*), and Ribosomal Protein S27a (*RPS27A*) (60). *UBC* is a known stress-regulated poly-ubiquitin gene which is upregulated during cellular stress as a protective response to provide additional ubiquitin and induce the degradation of unfolded or damaged proteins (61–63). RNA sequencing revealed that *UBC* gene expression was significantly upregulated in WT-infected cells compared to vehicle-treated cells (2.9-fold change, *padj* < 0.001), and this expression was diminished following infection with strains lacking *ECE1* or the candidalysin-encoding region. The expression of *UBC* was restored when epithelial cells were infected with an *ECE1* re-integrant strain (2.8-fold change, *padj* < 0.001) (Fig. 1C). Consistently, *UBC* gene expression was significantly increased in candidalysin-treated cells compared to control cells at both 2 and 6 h (*padj* < 0.001) (Fig. 1D). However, no change in *UBB*, *UBA52*, or *RPS27A* expression was observed following stimulation with *C. albicans* or candidalysin. These data suggest that the ubiquitin pool may be increased as a stress response to *C. albicans* infection, in a candidalysin-dependent manner.

Ubiquitin-specific proteases (USPs) are the largest family of de-ubiquitinating enzymes (DUBs) that regulate protein stability by reversing ubiquitination. Given their role in cellular homeostasis and signaling, we quantified USP expression during infection of oral epithelial cells. Several USPs were upregulated in response to *C. albicans* and candidalysin (Fig. 1C and D), including *USP36* (2.0-fold change, *padj* < 0.001) and *USP17L1* (1.9-fold change, *padj* < 0.01). In contrast, *USP13* was modestly but significantly downregulated during infection (0.6-fold change, *padj* < 0.01).

Tumor necrosis factor alpha-induced protein 3 (TNFAIP3, also called A20) is an essential ubiquitin-editing protein and a key negative regulator of the NF-κB pathway. TNFAIP3 functions both as a ubiquitin ligase and a de-ubiquitinase, playing a critical role in modulating inflammation (64). Notably, *TNFAIP3* was highly upregulated in response to both WT *C. albicans* (10.0-fold change, *padj* < 0.001) (Fig. 1C) and candidalysin (5.9-fold change, *padj* < 0.001) (Fig. 1D), suggesting a potential host response aimed at attenuating inflammatory signaling during infection. In addition, other enzymes involved in the ubiquitin-proteasome system were upregulated during infection, such as ubiquitin-associated protein 1 (1.7-fold change, *padj* < 0.001), which plays a role in the proteasomal degradation of ubiquitinated cell-surface proteins, including EGFR (65). Finally, two *CBL* genes, which are specifically required for EGFR ubiquitination, exhibited changes in expression but only in response to candidalysin at 6 h (1.9- and 2.0-fold changes, respectively, for *CBLC* and *CBL*; *padj* < 0.001) (Fig. 1D). Collectively, these observations suggest that candidalysin induces the expression of specific genes that are central to the ubiquitin system.

Next, we investigated protein ubiquitination in response to infection. WT *C. albicans* (SC5314) significantly increased protein ubiquitination compared to vehicle-treated cells (Fig. 1E and F). In contrast, infection with *ece1*Δ/Δ and *als3*Δ/Δ mutant strains did not trigger the same degree of protein ubiquitination. Instead, they induced patterns similar to the vehicle-treated cells, showing no significant increase in protein ubiquitination (Fig. 1E and F). These data demonstrate that *C. albicans* induces widespread ubiquitination of epithelial proteins and is a response driven by *ECE1* and *ALS3*.

### *C. albicans* induces EGFR ubiquitination in an *ECE1*-driven manner

**Fig 2:**
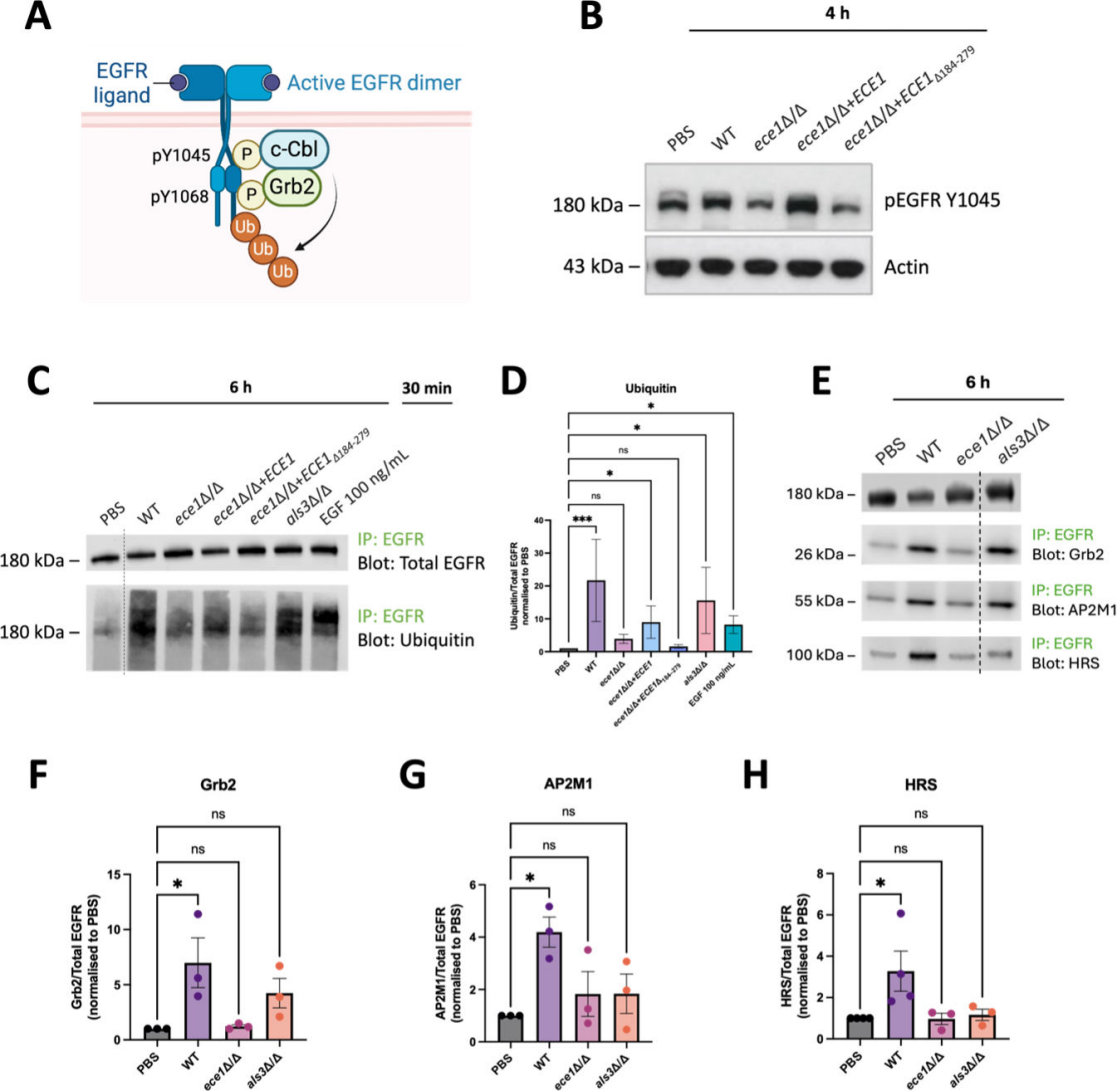
*C. albicans* induces EGFR phosphorylation at Y1045 and EGFR ubiquitination in TR146 oral epithelial cells in an *ECE1*-driven manner. (**A**) Schematic of EGFR phosphorylation and ubiquitination. Created with BioRender.com. (**B**) TR146 cells were infected with wild-type (WT) *C. albicans* (BWP17), an *ECE1* null strain (*ece1*Δ/Δ), an *ECE1* re-integrant strain (*ece1*Δ/Δ + *ECE1*), or a candidalysin null strain (*ece1*Δ/Δ + *ECE1*_Δ184–279_) for 4 h at an MOI of 10 or stimulated with PBS as a vehicle control. Lysates (10 µg) were electrophoresed on gradient gels to detect pEGFR Y1045 and α-actin via Western blot. Data are representative of three independent experiments. (**C**) TR146 cells were infected with WT *C. albicans* (SC5314), *ece1*Δ/Δ, *ece1*Δ/Δ + *ECE1*, *ece1*Δ/Δ+*ECE1*_Δ184–279_, and *als3*Δ/Δ for 6 h at an MOI of 5 or stimulated with PBS as a vehicle control. EGF 100 ng/mL was used as a positive control. Lysates were collected and EGFR was immunoprecipitated. Immunoprecipitation (IP) samples were electrophoresed on gradient gels to detect total EGFR and ubiquitin via Western blot. Data are representative of three independent experiments. Dashed vertical lines indicate omitted, extraneous portions of blot images. (**D**) Relative protein levels in panel **C** were quantified using densitometry. Ubiquitin was normalized to total EGFR and expressed relative to the control (PBS), which was set to 1. Data represent the average of three to seven independent experiments and are presented as mean values with standard error of the mean (SEM) error bars. Statistical significance was determined using the Kruskal–Wallis test with Dunn’s multiple comparison test. *P* < 0.05, ***P < 0.001. (**E**) TR146 cells were infected with WT *C. albicans* (SC5314), *ece1*Δ/Δ, and *als3*Δ/Δ for 6 h at an MOI of 5 or stimulated with PBS as a vehicle control. Lysates were collected and EGFR was immunoprecipitated. IP samples were electrophoresed on gradient gels to detect total EGFR, Grb2, AP2M1, and HRS via Western blot. Dashed vertical lines indicate omitted, extraneous portions of blot images. Data are representative of three independent experiments. (**F–H**) Relative protein levels in panel E were quantified using densitometry. Grb2, AP2M1, and HRS were normalized to total EGFR and expressed relative to the control (PBS), which was set to 1. Data represent the average of three to four independent experiments and are presented as mean values with SEM error bars. Statistical significance was determined using a parametric test (ordinary one-way ANOVA with Dunnett’s multiple comparison test). *P < 0.05. ns, not significant.

Since *C. albicans* infection induced the ubiquitination of numerous epithelial proteins, we next investigated whether EGFR itself was ubiquitinated. Upon activation, EGFR undergoes phosphorylation at multiple sites, including tyrosine 1068, which is known to be phosphorylated in response to candidalysin (25, 55). However, phosphorylation of tyrosine 1045 (Y1045) is also crucial for EGFR ubiquitination and degradation (39). Phosphorylation at Y1045 creates a docking site for c-Cbl, an E3 ubiquitin ligase responsible for EGFR ubiquitination. c-Cbl can also be recruited to EGFR indirectly through the adaptor protein, Grb2 (37, 66, 67) (Fig. 2A). Accordingly, we investigated whether *C. albicans* could induce phosphorylation of EGFR at Y1045 in TR146 cells. Robust phosphorylation of EGFR at Y1045 was observed in response to WT *C. albicans* at 4 h, which was markedly reduced in response to strains lacking *ECE1* or the candidalysin-encoding region (Fig. 2B).

To investigate EGFR ubiquitination, we employed an immunoprecipitation (IP) assay. Despite lower levels of immunoprecipitated EGFR, WT *C. albicans* induced significant levels of EGFR ubiquitination compared to the PBS control, as shown by the high molecular weight smear from ~180 kDa and above (Fig. 2C and D). Cells infected with an *als3*Δ/Δ mutant also exhibited strong EGFR ubiquitination. In contrast, the *ece1*Δ/Δ and candidalysin null strains were unable to induce significant receptor ubiquitination compared to the PBS control (Fig. 2C and D). Ubiquitination was restored in response to infection with an *ECE1* re-integrant strain, showing that EGFR ubiquitination is driven by *ECE1*. Stimulation of epithelial cells with EGF (positive control) induced robust EGFR ubiquitination as expected (34, 68).

### Grb2 recruitment to EGFR in response to *C. albicans* is *ECE1* and *ALS3* driven

To better understand the molecular players involved in EGFR activation, we next examined the interaction of EGFR with key adaptor proteins that may mediate its trafficking and turnover. EGFR ubiquitination is facilitated by several adaptor proteins, with the E3 ubiquitin-protein ligase c-Cbl playing a central role in this process (37, 39). As mentioned, c-Cbl directly interacts with phosphorylated Y1045 on EGFR (39, 41) and can be recruited to EGFR indirectly through the adaptor protein Grb2 (37, 66, 67), an essential “missing link” between EGFR and Ras-MAPK signaling (69) (Fig. 2A).

We previously demonstrated that *C. albicans* and candidalysin induced the activation of both c-Cbl and Grb2 (70), and *C. albicans* can induce EGFR phosphorylation at Y1045 and EGFR ubiquitination (Fig. 2A through D). To investigate whether c-Cbl and Grb2 interact directly with EGFR during infection, we performed co-IP studies. Infection of TR146 cells with WT *C. albicans* revealed that Grb2 directly interacts with EGFR during infection (Fig. 2E and F). In contrast, c-Cbl showed only a weak and inconsistent interaction with EGFR, intermittently detectable near the assay’s sensitivity limit (L. Lortal, unpublished observations). These findings demonstrate that Grb2 is recruited to EGFR and may be required to mediate EGFR ubiquitination and subsequent signaling events in response to fungal infection. We next addressed whether *C. albicans-*induced recruitment of Grb2 was driven by Ece1p or Als3p. The *ece1*Δ/Δ and *als3*Δ/Δ strains did not induce any significant recruitment of Grb2 to EGFR (Fig. 2E and F), suggesting that Grb2 recruitment to EGFR is driven by both *ECE1* and *ALS3*.

### *C. albicans* induces increased interaction of EGFR with AP2M1 and HRS

After ubiquitination, EGFR is normally internalized into the cell. Clathrin-mediated endocytosis is an early and major pathway for receptor internalization and is initiated by the binding of clathrin adaptors, such as adaptor protein complex 2 (AP2). Once internalized and in early endosomes, EGFR can follow two different routes: degradation or recycling back to the plasma membrane (32–35).

To investigate early events of clathrin-mediated endocytosis in *C. albicans*-induced EGFR trafficking, we examined the interaction between EGFR and AP2M1, a subunit of the AP2 complex, which labels EGFR for internalization (35, 71). While there was a basal interaction between EGFR and AP2M1 observed in vehicle-treated cells, infection with WT *C. albicans* significantly increased the association of AP2M1 with EGFR (Fig. 2E and G). Notably, infection with *ece1*Δ/Δ or *als3*Δ/Δ did not significantly increase the AP2M1-EGFR interaction (Fig. 2E and G), suggesting *C. albicans* induces EGFR endocytosis in an *ECE1*- and *ALS3*-dependent manner.

We next examined later events of EGFR trafficking by investigating the interaction between EGFR and HRS, a component of the endosomal sorting complex required for transport machinery. This interaction sorts ubiquitinated EGFR into intraluminal vesicles of multivesicular bodies for subsequent lysosomal degradation (35, 38). Like AP2M1, HRS strongly interacted with EGFR following infection with WT *C. albicans* but not with mutant strains lacking *ECE1* or *ALS3* (Fig. 2E and H).

Together, these data suggest that during *C. albicans* infection, Ece1p and Als3p induce the recruitment of EGFR adaptor proteins Grb2, AP2M1, and HRS, which are important for EGFR ubiquitination, endocytosis, and trafficking.

### *C. albicans* promotes EGFR degradation through Ece1p and Als3p, predominantly mediated by the lysosomal pathway

**Fig 3:**
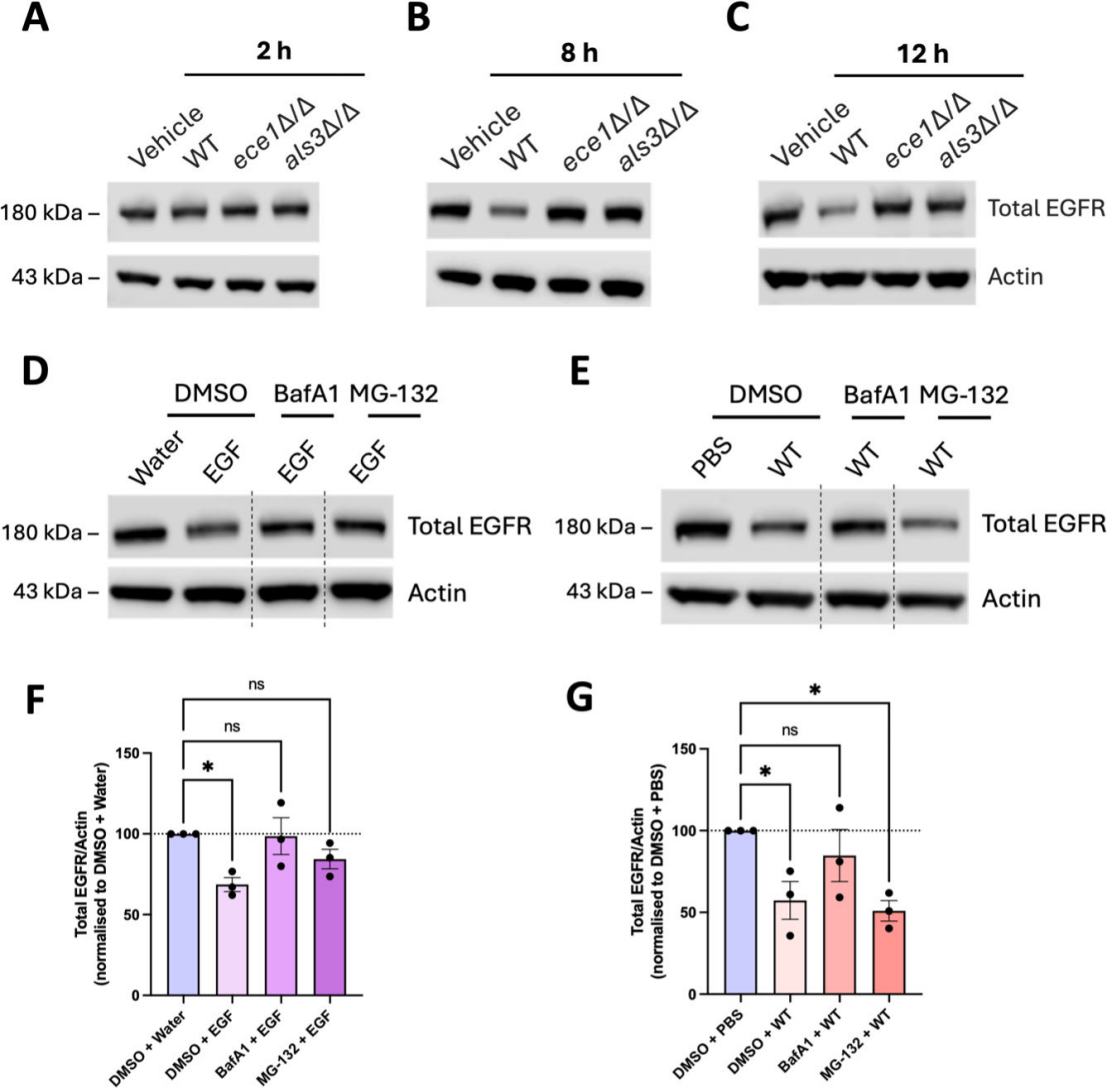
*C. albicans* promotes EGFR degradation through Ece1p and Als3p, predominantly mediated by the lysosomal pathway, and induces the recruitment of adaptor proteins Grb2, AP2M1, and HRS. (**A–C**) TR146 cells were infected with different *C. albicans* strains (wild-type [WT] SC5314, *ece1*Δ/Δ and *als3*Δ/Δ) or stimulated with PBS as a vehicle control for 2 h (MOI 10), 8 h (MOI 1), and 12 h (MOI 1). Cell lysates were electrophoresed on gradient gels to detect total EGFR and α-actin via Western blot. Data are representative of three independent experiments. (**D and E**) TR146 cells were pre-treated for 1 h with 0.25 µM of lysosomal inhibitor Bafilomycin A1 (BafA1) and 10 µM of proteasomal inhibitor MG-132 or DMSO as a vehicle control, and then treated with 100 ng/mL EGF as a positive control for 1 h or infected with SC5314 (MOI 5) for 10 h. Cell lysates were electrophoresed on gradient gels to detect total EGFR and α-actin via Western blot. Data are representative of three independent experiments. Dashed vertical lines indicate omitted, extraneous portions of blot images. (**F and G**) Relative protein levels in panels **D and E** were quantified using densitometry. Total EGFR was normalized to α-actin and expressed relative to the control (DMSO + water or DMSO + PBS), which was set to 100. Data are presented as mean values with standard error of the mean error bars. Statistical significance was determined using a parametric test (ordinary one-way ANOVA with Dunnett’s multiple comparison test). *P < 0.05. ns, not significant.

We next examined receptor fate by assessing total EGFR levels. At 2 h post-infection, total EGFR levels remained stable regardless of the infecting strain (Fig. 3A). However, at later time points (6, 8, 10, and 12 h), cells infected with WT *C. albicans* exhibited a decrease in total EGFR levels, indicating EGFR degradation over time (Fig. 3B and C; Fig. S1A and B). In contrast, infection with *ece1*Δ/Δ or *als3*Δ/Δ mutants failed to induce similar EGFR degradation, suggesting that *C. albicans* drives this process through Ece1p and Als3p.

To further investigate the mechanism underpinning EGFR degradation, we inhibited both lysosomal and proteasomal activity to assess their contribution to the observed receptor loss. TR146 cells were pre-treated with 0.25 µM of lysosomal inhibitor Bafilomycin A1 (BafA1) or 10 µM of proteasomal inhibitor MG-132, both previously shown to inhibit EGF-induced EGFR degradation (72, 73). Cells were then treated with 100 ng/mL EGF or infected with WT *C. albicans*. As expected, in cells pre-treated with the DMSO vehicle control, WT *C. albicans* and EGF led to EGFR degradation, with mean EGFR levels reduced to 68.5% and 57.3% of baseline (DMSO + PBS or DMSO + water), respectively, based on densitometry analysis (Fig. 3D through G). The use of either inhibitor almost fully rescued EGF-induced EGFR degradation (to 98.6% with BafA1 and 84.4% with MG-132), demonstrating that EGF-induced EGFR degradation is host-driven through both canonical lysosomal and proteasomal degradation pathways (Fig. 3D and F). In contrast, in *C. albicans*-infected cells, BafA1 treatment rescued EGFR degradation (up to 84.8%), while MG-132 pre-treated cells still exhibited EGFR degradation (down to 51%) (Fig. 3E and G). Collectively, these data demonstrate that *C. albicans* promotes EGFR degradation through Ece1p and Als3p in a mechanism that is predominantly mediated by the lysosomal pathway.

### *Egfr* expression is upregulated in an oropharyngeal candidiasis mouse model

**Fig 4:**
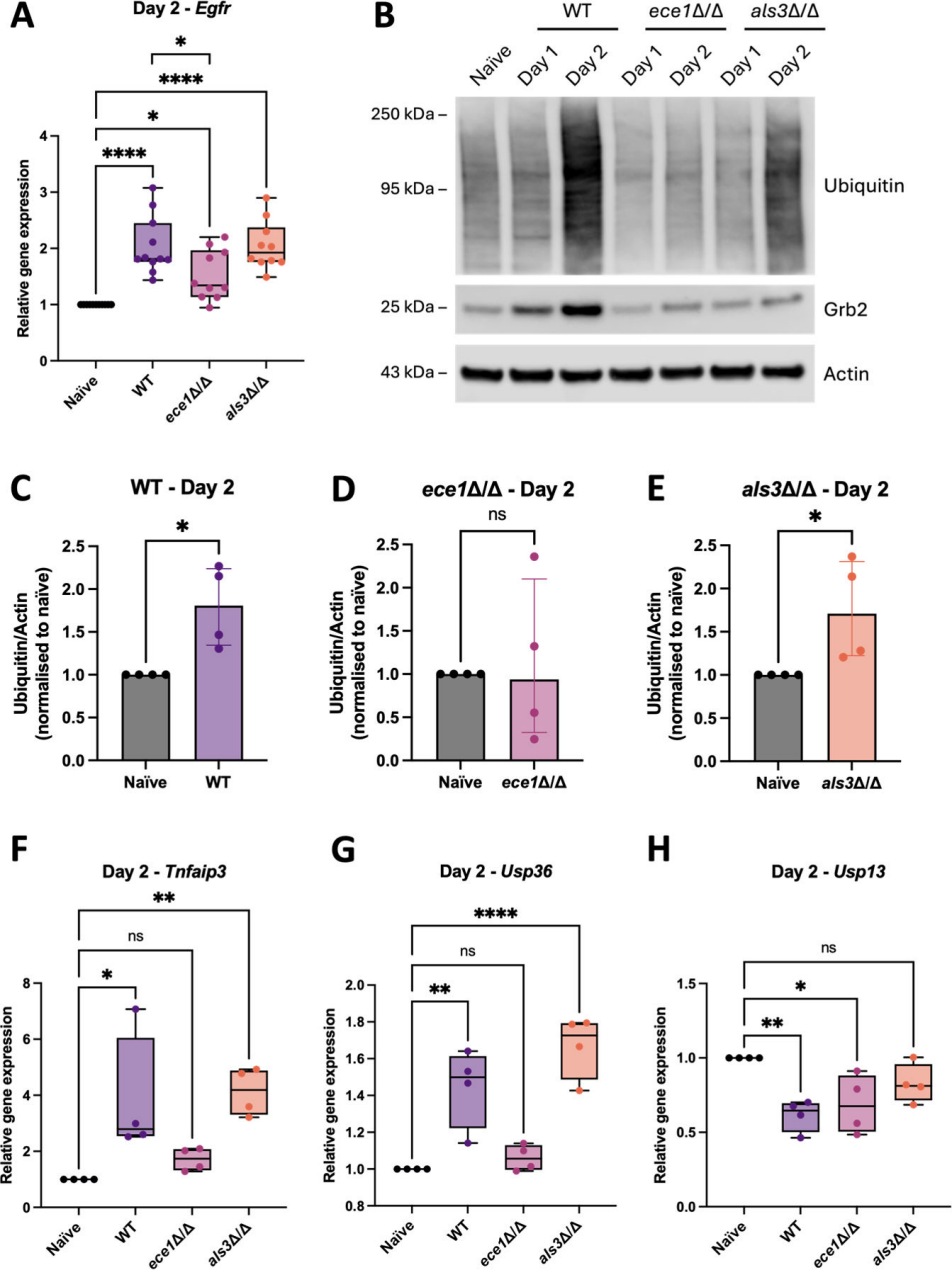
*C. albicans* dynamically regulates *Egfr*, ubiquitin pathway-associated genes, and host protein ubiquitination in an oropharyngeal candidiasis mouse model. C57BL/6J mice were sublingually infected with wild-type (WT) *C. albicans* AHY940 and mutant strains *ece1*Δ/Δ and *als3*Δ/Δ, and tongues were harvested at 24 and 48 h post-infection. Gene expression of (**A**) *Egfr*, (**F**) *Tnfaip3*, (**G**) *Usp36*, and (**H**) *Usp13* was quantified using ΔΔCt relative to the naïve control. Data were collected from one to three independent experiments with three to four mice/group. Data are plotted as box plots, where the central line represents the median; the box indicates the interquartile range; and the whiskers extend to the minimum and maximum values. Individual data points represent biological replicates. A Shapiro–Wilk test was first used to determine data normality. If data were normal, a parametric test (ordinary one-way ANOVA with Dunnett’s multiple comparison test) was performed. If data were not normal, a non-parametric test (Kruskal–Wallis test with Dunn’s multiple comparison test) was used to determine statistical significance. An unpaired *t*-test was used for pairwise comparisons between specific strains. (**B**) Tongue lysates were electrophoresed on gradient gels and analyzed by Western blot to detect the presence of ubiquitin and Grb2. α-Actin was used as a loading control. Data are representative of three independent experiments. (**C–E**) Relative protein levels in panel **B** were quantified using densitometry. Target protein levels were normalized to actin and expressed relative to the naïve control, which was set to 1. Data are presented as median with interquartile range. Individual data points represent biological replicates. Statistical significance was determined using an unpaired non-parametric *t*-test (Kolmogorov–Smirnov test). *P < 0.05, **P < 0.01, ****P < 0.0001. ns, not significant.

Given the importance of EGFR in *C. albicans* infection *in vitro*, we next investigated *Egfr* gene expression *in vivo*. Using an OPC mouse model, we assessed *Egfr* expression in tongue tissue at different time points (days 1 and 2) in response to WT *C. albicans* (AHY940, derivative of SC5314) and *ece1*Δ/Δ and *als3*Δ/Δ mutant strains (constructed via CRISPR/Cas9, as described by Nguyen et al. [74]). On day 1, *Egfr* gene expression remained stable in response to all strains compared to the naïve control, indicating no immediate induction of gene expression following infection (Fig. S2A). However, by day 2, *Egfr* expression was significantly upregulated in response to WT *C. albicans* and *als3*Δ/Δ. *Egfr* upregulation was significantly reduced in response to *ece1*Δ/Δ (Fig. 4A), indicating that *ECE1* predominantly drives EGFR-related host responses during OPC.

### *C. albicans* dynamically regulates ubiquitin pathway-associated genes and protein ubiquitination *in vivo*

We next examined the expression of ubiquitin and ubiquitin pathway-associated genes *in vivo*. Analysis of *UbC* gene expression revealed no significant changes between WT *C. albicans* and mutant strains compared to the naïve control at either day 1 or day 2 (Fig. S2B and C). However, notably, protein ubiquitination in the whole tongue homogenate was significantly increased on day 2 in response to both WT *C. albicans* and *als3*Δ/Δ compared to the naïve control, but not in response to *ece1*Δ/Δ (Fig. 4B through E). These findings suggest that while *UbC* gene expression remains stable, the regulation of ubiquitination at the protein level is dynamic and *ECE1* driven.

We also assessed the protein levels of the EGFR adaptor protein Grb2. On day 1, Grb2 protein levels were significantly increased in response to WT *C. albicans* only (Fig. 4B; Fig. S2D through F). By day 2, *ece1*Δ/Δ and *als3*Δ/Δ also induced an increase in Grb2 protein levels compared to naïve but which remained lower than with WT *C. albicans* (Fig. 4B; Fig. S2G through I), suggesting a delayed response to infection with the *ece1*Δ/Δ and *als3*Δ/Δ mutants. The data suggest that, like *in vitro*, Grb2 plays a role during infection *in vivo*.

In addition to *UbC*, we examined the expression of three other genes that were upregulated or downregulated during *C. albicans* infection *in vitro. Tnfaip3* gene expression was significantly upregulated in response to both WT and *als3*Δ/Δ but not *ece1*Δ/Δ at both days 1 and 2 (Fig. 4F; Fig. S2J). Similarly, significant upregulation of *Usp36* was detected on day 2 (not day 1) in response to WT and *als3*Δ/Δ, but not *ece1*Δ/Δ (Fig. 4G; Fig. S2K). Interestingly, expression of *Usp13* was downregulated in response to all strains on day 1, with the most significant reduction observed with WT *C. albicans* (Fig. S2L), which persisted to day 2 (Fig. 4H). Together, these data reveal that ubiquitin pathway-associated genes (*UbC*, *Tnfaip3*, *Usp36*, and *Usp13*) are distinctly regulated during *C. albicans* OPC infection and underscore the importance of *ECE1* in modulating pathways related to ubiquitination *in vivo*.

### EGFR cKO is protective during wild-type *C. albicans* infection

To further investigate the role of EGFR during oral *C. albicans* infection, a tamoxifen-inducible EGFR cKO mouse model was generated, in which EGFR was specifically deleted in K14 tissues (only active in squamous epithelial tissues, including the oral cavity) (Fig. S3A). The use of tamoxifen to induce EGFR deletion permits the modeling of an EGFR cKO, as mice with a constitutive EGFR KO are not viable. This powerful model also avoids potential systemic effects and permits the precise evaluation of *Egfr* gene function in OPC. EGFR deletion in K14 tissue only was confirmed by histological analysis (Fig. S3B and C).

**Fig 5:**
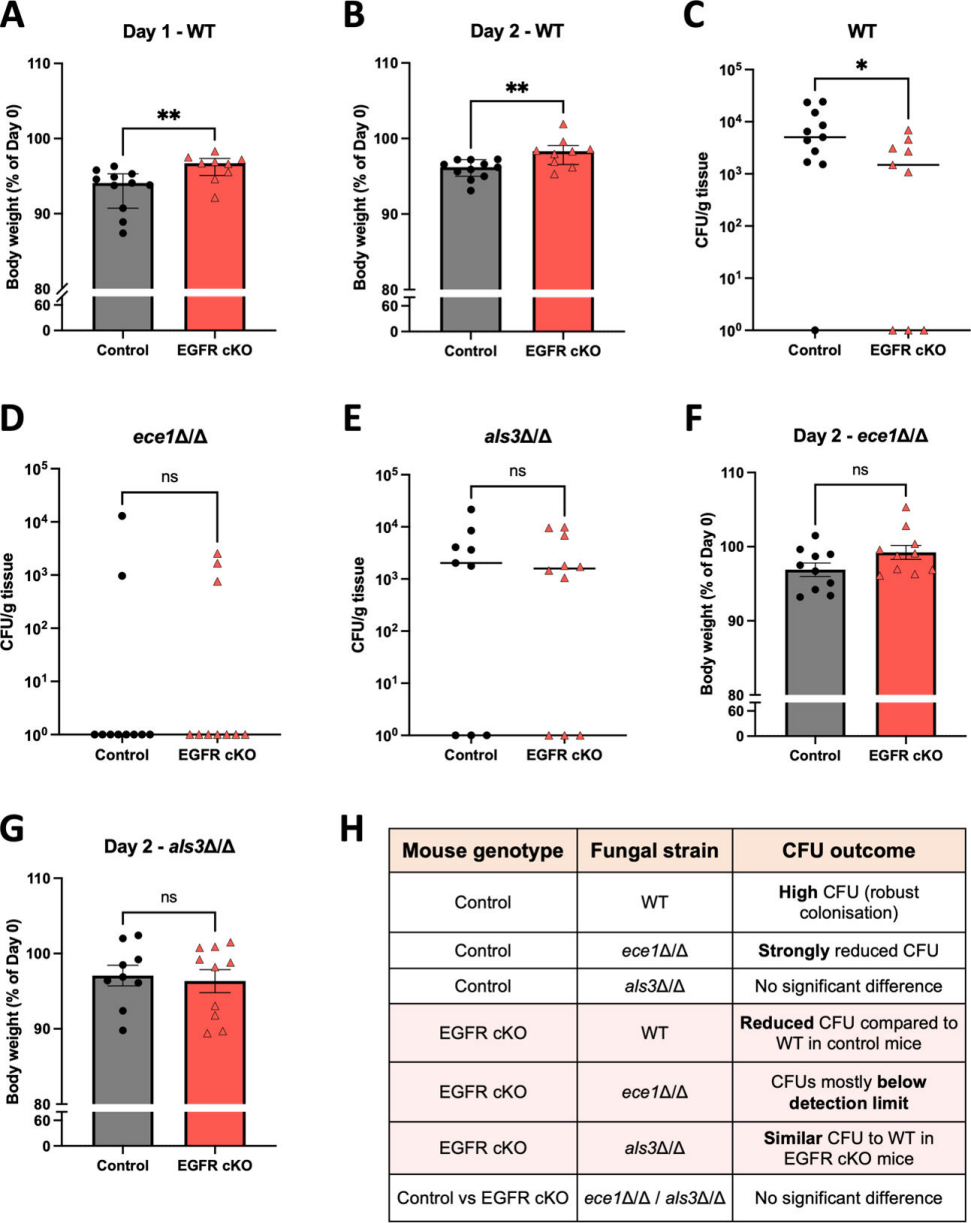
EGFR cKO mice display significantly lower fungal burden and less weight loss than control mice in response to wild-type (WT) *C. albicans* infection. EGFR cKO (EGFR^fl/fl^ K14^Cre+^) and control (EGFR^fl/fl^ K14^Cre−^) mice were sublingually infected with WT *C. albicans* AHY940 and mutant strains *ece1*Δ/Δ and *als3*Δ/Δ. (**A and B, F and G**) Mice were weighed at days 0, 1, and 2 and plotted as a percent weight of day 0. A Shapiro–Wilk test was first used to determine data normality. Day 1 weight data for WT were non-normally distributed and plotted as median values with interquartile range error bars. All day 2 weight data were normally distributed and plotted as mean values with standard error of the mean error bars. If data were normal, a parametric test (unpaired *t*-test) was performed. If data were not normal, a non-parametric test (Mann–Whitney test) was used to determine statistical significance. (**C–E**) Tongues were harvested on day 2 post-infection to measure colony-forming units (CFU). Data were transformed by *Y* = *Y* + *K*, *K* = 1. CFU data were plotted on a log scale where the central line represents the median. Individual data points represent biological replicates. *P* < 0.05, **P < 0.01. Data were collected from three experiments with three to four mice/group. (**H**) Table summarizing relative fungal burdens observed in control and EGFR cKO mice following oral infection with WT, *ece1*Δ/Δ, or *als3*Δ/Δ *C. albicans* strains. ns, not significant.

We infected EGFR^fl/fl^ K14^Cre+^ (EGFR cKO) and EGFR^fl/fl^ K14^Cre−^ (control) mice with WT *C. albicans* (AHY940), *ece1*Δ/Δ and *als3*Δ/Δ and quantified fungal burden (CFU), weight, and expression of cytokines and chemokines. *ECE1* gene expression was confirmed in AHY940 via quantitative PCR (qPCR) (Fig. S3D). EGFR cKO mice infected with WT *C. albicans* experienced significantly reduced weight loss compared to control mice on days 1 and 2 (Fig. 5A and B). EGFR cKO mice also displayed significantly lower fungal burden at day 2 (termination) (Fig. 5C). Indeed, WT *C. albicans* was cleared more readily in EGFR cKO mice, with 3 of 9 animals (33.3%) showing no detectable fungal burden, compared to 1 of 11 control mice (9.1%).

In control mice, infection with *als3*Δ/Δ resulted in a higher proportion of animals with undetectable fungal burden (33.3% vs 9.1%), but this difference was not statistically significant, while fungal burden was significantly reduced with *ece1*Δ/Δ (Fig. S3E). However, in EGFR cKO mice, infection with *ece1*Δ/Δ or *als3*Δ/Δ resulted in fungal burdens statistically comparable to those observed with WT *C. albicans* (Fig. S3F). Notably, infection with either mutant strain yielded no significant difference in CFU levels and body weights in EGFR cKO and control mice (Fig. 5D through G). Indeed, with *ece1*Δ/Δ, fungal burdens were mostly below the detection limit in both groups (8/10 in control mice and 7/10 mice in EGFR cKO mice), suggesting failure to establish infection and/or rapid clearance (Fig. 5D). However, while fungal burden was still present in *als3*Δ/Δ-infected mice, EGFR cKO had no impact on CFU count (Fig. 5E). A summary of all outcomes is presented in Fig. 5H. Together, the data suggest that *ECE1* may contribute to colonization in an EGFR-independent manner, while *ALS3* requires EGFR to exert its colonization-promoting effects.

**Fig 6:**
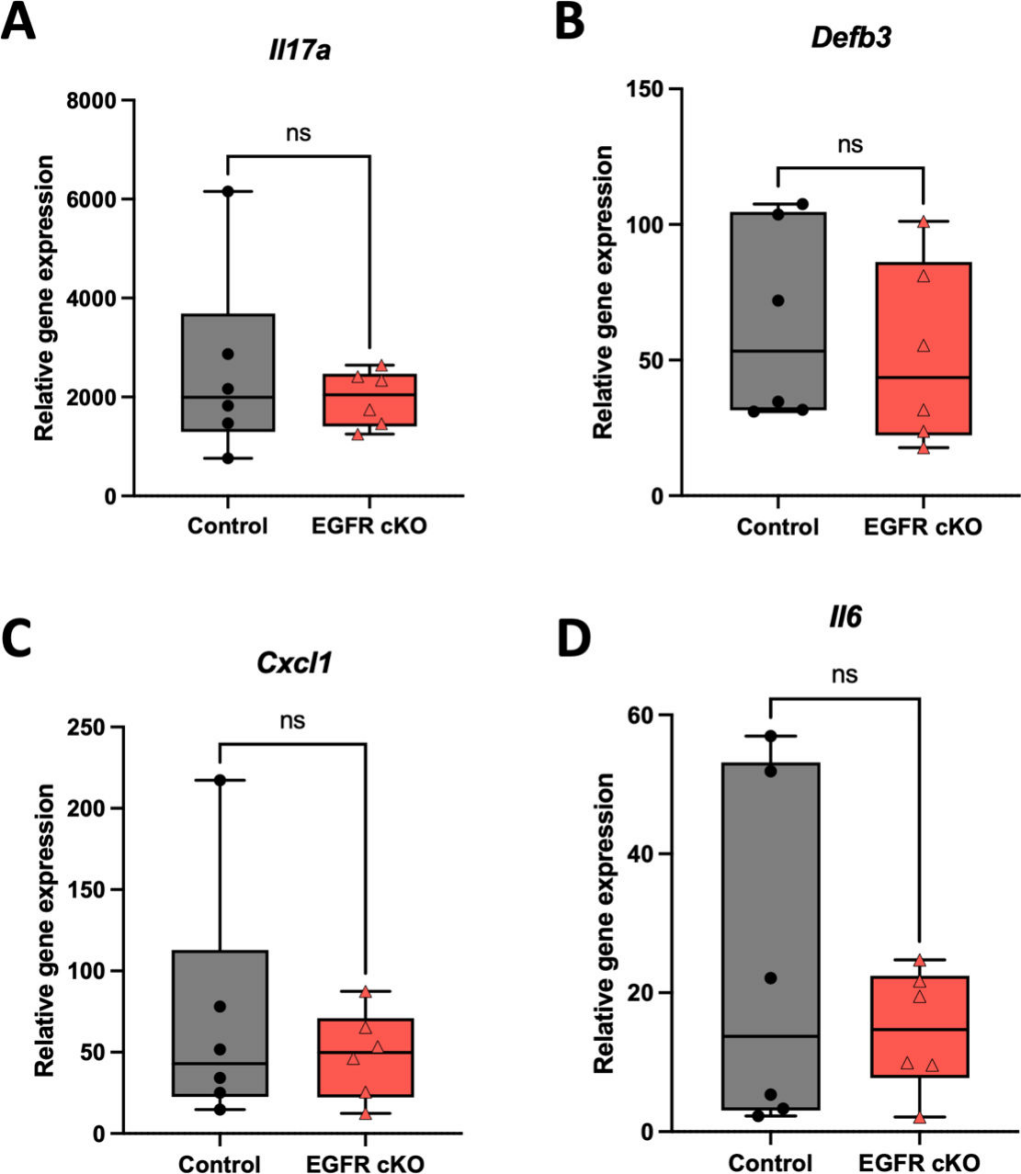
Mucosal immunity to *C. albicans* infection is not significantly impaired in EGFR cKO mice at day 2. EGFR cKO (EGFR^fl/fl^ K14^Cre+^) and control (EGFR^fl/fl^ K14^Cre−^) mice were sublingually infected with wild-type *C. albicans* AHY940. Gene expression of (**A**) *Il17a*, (**B**) *Defb3*, (**C**) *Cxcl1*, and (**D**) *Il6* at day 2 was quantified using ΔΔCt relative to the naive control. Data are plotted as box plots, where the central line represents the median; the box indicates the interquartile range; and the whiskers extend to the minimum and maximum values. Individual data points represent biological replicates. A Shapiro–Wilk test was first used to determine data normality. If data were normal, a parametric test (unpaired *t*-test) was performed. If data were not normal, a non-parametric test (Mann–Whitney test) was used to determine statistical significance. Data were collected from one to three independent experiments with three to four mice/group. ns, not significant.

Given that *C. albicans* robustly induces inflammatory responses during OPC (17, 22, 24, 25, 28, 55) and EGFR promotes *C. albicans* infection (Fig. 5A through C), we asked whether epithelial EGFR deletion alters cytokine gene induction. We quantified transcripts for known *C. albicans*-induced inflammatory markers (*Il17a*, *Il1b*, *Cxcl1*, *Defb3*, and *Il6*) in control and EGFR cKO mice following infection with WT *C. albicans*. Aside from moderately increased *Il17a* expression in control mice at day 1, cytokine gene expression was comparable between control and EGFR cKO mice at days 1 and 2 (Fig. 6; Fig. S4). Thus, EGFR loss in K14+ oral epithelium does not measurably reduce induction of these cytokines, despite the reduced fungal burden observed at day 2. These findings support a model in which K14+ epithelial EGFR primarily facilitates tissue colonization, whereas cytokine gene induction reaches similar levels via EGFR-independent sensing pathways during OPC.

## DISCUSSION

In this study, we demonstrate that *C. albicans* promotes EGFR ubiquitination and its subsequent degradation in oral epithelial cells, predominantly through the lysosomal pathway and in an Ece1p- and Als3p-dependent manner. We further reveal broad changes in cellular protein ubiquitination and expression of ubiquitin pathway-associated genes in response to *C. albicans* infection, both *in vitro* and *in vivo.* Finally, we show that during OPC, EGFR cKO is protective but might not be critical in driving the immune response.

In human oral epithelial cells, *C. albicans* and candidalysin induced upregulation of several EGFR ligands, including *EREG*, *AREG*, *EPGN*, and *HBEGF*, while EGFR mRNA levels remained largely unchanged. In contrast, in a mouse model of OPC, we observed significant upregulation of *Egfr* during OPC, predominantly driven by Ece1p. This suggests a divergence between human epithelial cell-intrinsic and mouse tissue-level responses, with host tissues potentially compensating for fungal-driven EGFR degradation by enhancing transcriptional upregulation of EGFR.

We further demonstrate that *C. albicans* induces EGFR ubiquitination in oral epithelial cells in an Ece1p-driven manner. Ubiquitination of EGFR is a well-characterized post-translational modification that typically regulates receptor internalization and degradation. While EGFR ubiquitination has been studied in response to viral pathogens (e.g., the hepatitis C virus non-structural protein 5A blocks EGFR degradation through the loss of EGFR ubiquitination [52]), its role in modulating host responses to fungal infection has remained unexplored. Our findings indicate that *C. albicans* actively induces EGFR ubiquitination through Ece1p, potentially representing a pathogen-driven immune evasion strategy aimed at dampening EGFR-mediated ERK1/2 signaling, cytokine production, and subsequent type 17 immune responses that are critical for fungal clearance.

We observed direct recruitment of Grb2, AP2M1, and HRS to EGFR during *C. albicans* infection, indicating activation of canonical endocytic and sorting pathways. Numerous proteins interact with EGFR during its trafficking, influencing its fate (75, 76). A recent study in OKF6/TERT-2 oral epithelial cells identified over 1,000 EGFR-associated proteins following *C. albicans* infection for 90 min (77), providing a comprehensive snapshot of early EGFR trafficking events during infection. Our study complements these findings by confirming key EGFR interactions, specifically with Grb2, AP2M1, and HRS, in TR146 oral epithelial cells after 6 h of infection, capturing later-stage trafficking dynamics relevant to sustained host–pathogen interactions. Notably, Phan et al. (77) found Grb2, AP2A1, and AP2B1 (subunits of the AP2 complex) to interact with EGFR. AP2M1 was identified in one of the three replicates, but neither c-Cbl nor HRS was identified. Our targeted validations reinforce the biological relevance of specific EGFR-associated proteins identified in large-scale interactome studies and suggest that key EGFR interactions are conserved across epithelial models and time points. The observed upregulation of total Grb2 in mouse tongue tissue during OPC further supports a potential host-driven mechanism to enhance EGFR signaling.

Although we demonstrate that *C. albicans* induces EGFR ubiquitination, the consequences of this modification for fungal uptake remain less clear. Direct pharmacological inhibition of EGFR ubiquitination is currently not feasible, as there are no selective approaches beyond targeting its E3 ligase, c-Cbl. Notably, knockdown of c-Cbl or Grb2, both required for efficient EGFR ubiquitination, did not alter epithelial damage (70). This suggests that these adaptors, and by extension EGFR ubiquitination, do not influence epithelial damage and may have limited impact on *C. albicans* endocytosis, since formation of the invasion pocket is critical for candidalysin-mediated damage (18). Nevertheless, the possibility that EGFR ubiquitination contributes to endocytic uptake of *C. albicans* remains an important question for future investigation.

Total EGFR levels remained stable *in vitro* at early time points (2 h), consistent with a study showing stable total EGFR levels after 30 and 90 min of *C. albicans* infection (55). However, we observed EGFR degradation at later time points (from 6 h onward), driven by candidalysin and Als3p, which occurred predominantly via the lysosomal pathway and was consistent with receptor ubiquitination and canonical degradation. While Als3p appears to be required for EGFR ubiquitination, adaptor recruitment, and EGFR degradation, its role is far less significant compared to Ecep1. The role of Als3p is likely to be indirect, as it is critical for epithelial adherence and invasion pocket formation, which facilitates the local accumulation of candidalysin at the epithelial surface (11, 15–18). In the absence of Als3p, reduced delivery and concentration of candidalysin to the pocket may attenuate the ability of *C. albicans* to trigger EGFR ubiquitination and downstream trafficking. This highlights the functional interplay between Als3p and Ece1p in orchestrating host–receptor manipulation.

Interestingly, EGFR trafficking in response to *C. albicans* mirrors that of innate immune receptors Dectin-2 and Dectin-3, which are both ubiquitinated by the E3 ubiquitin ligase Cbl-b in response to fungal ligands (78). Similar to our findings with EGFR, inhibition of the lysosomal but not proteasomal degradation blocked *C. albicans*-induced degradation of Dectin-2 and Dectin-3 (78). Furthermore, *Cbl-b*^−/−^ mice have enhanced survival and reduced weight loss during systemic candidiasis (78–80), implicating Cbl-b-mediated receptor degradation as a broader immune regulatory mechanism during *C. albicans* infection.

**Fig 7:**
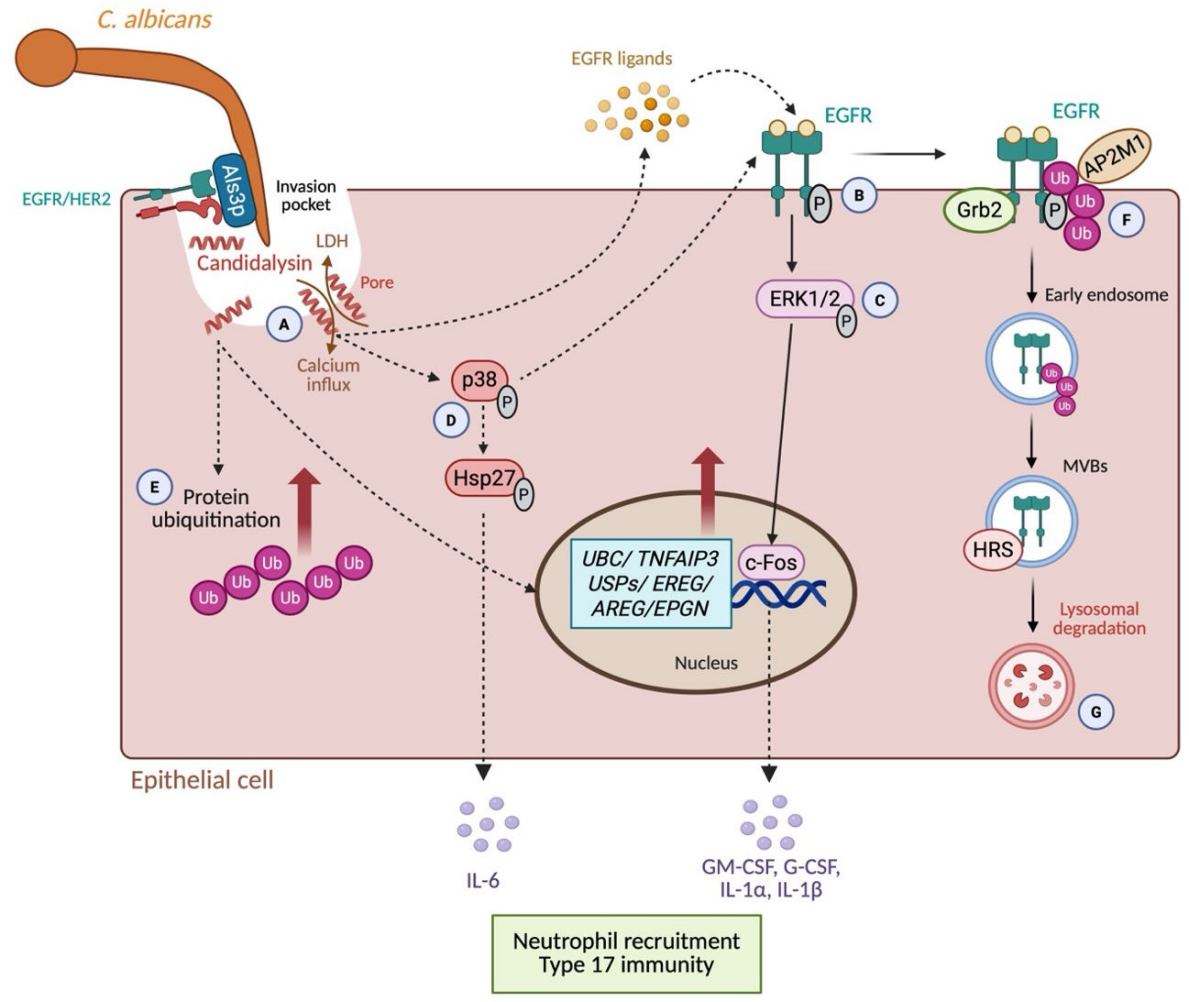
Model for epithelial cell activation and EGFR trafficking induced by *C. albicans*. (A) During epithelial infection, *C. albicans* hyphae form an invasion pocket, predominantly through the binding of the fungal adhesin/invasin Als3p to the EGFR/HER2 complex on epithelial cells. Hyphae secrete the cytolytic peptide toxin candidalysin, which accumulates in the invasion pocket prior to forming pores in the plasma membrane, triggering calcium influx and cell damage (LDH release). Calcium influx stimulates the activation of matrix metalloproteinases, which release surface-tethered epidermal growth factor receptor (EGFR) ligands, epigen (EPGN), epiregulin (EREG), and amphiregulin (AREG). (B) These ligands bind to EGFR, triggering receptor dimerization and activation. (C) EGFR activation induces ERK1/2 phosphorylation and the subsequent activation of the transcription factor c-Fos. This leads to the expression of cytokines (G-CSF, GM-CSF, IL-1α, and IL-1β), which are released from the cell, inducing neutrophil recruitment and type 17 immunity. (D) In parallel, candidalysin activates the p38-MAPK pathway, which can also activate EGFR and Hsp27 and controls IL-6 secretion. (E) Candidalysin also drives the upregulation of ubiquitin C (*UBC*) and ubiquitin pathway-associated genes such as *USPs* and *TNFAIP3*, as well as an increase in total protein ubiquitination. (F) Once EGFR is activated, adaptor protein Grb2 is recruited, and EGFR is ubiquitinated. (G) EGFR is then internalized into early endosomes, trafficked into multivesicular bodies (MVBs), and finally degraded in lysosomes. The degradation of EGFR negatively regulates EGFR signaling. This model is based on *in vitro* data. Figure created with BioRender.com.

Our model for epithelial cell activation and EGFR trafficking induced by *C. albicans* is shown in Fig. 7. Our *in vitro* results, combined with previous studies, suggest a model in which candidalysin secretion results in calcium influx, EGFR ligand release, as well as transcriptional upregulation of EGFR ligands and components of the host ubiquitination machinery (Fig. 7A), followed by EGFR phosphorylation (Fig. 7B). This leads to activation of the ERK1/2-MAPK pathway, downstream signaling, and cytokine release (Fig. 7C). In parallel, candidalysin also activates the p38-MAPK pathway (Fig. 7D). This is accompanied by increased whole-cell ubiquitination, which may reflect both EGFR-specific and broader host responses (Fig. 7E). EGFR is then ubiquitinated, coinciding with recruitment of adaptor proteins such as Grb2, AP2M1, and HRS, which are involved in receptor internalization and trafficking (Fig. 7F). EGFR is subsequently degraded through the endolysosomal pathway (Fig. 7G). Together, these results identify EGFR ubiquitination as a central regulatory step that connects early ligand-driven activation to downstream receptor downregulation and degradation during *C. albicans* infection. Notably, both *ECE1* and *ALS3* are required to maximize EGFR ubiquitination.

*In vitro* experiments were conducted using *C. albicans* SC5314, which is commonly used in pathogenicity studies. While SC5314 provides a well-characterized model, we acknowledge that strain-specific differences in pathogenicity and host interactions exist among clinical isolates. Future studies comparing multiple isolates of varying pathogenicity will be valuable to determine whether the EGFR ubiquitination and trafficking effects described here are broadly conserved.

Although *C. albicans* and Ecep1 drive EGFR degradation and epithelial damage *in vitro*, the upregulation of *Egfr* mRNA during OPC suggests a host compensatory mechanism aimed at preserving EGFR signaling and supporting subsequent fungal clearance. In our OPC model, EGFR cKO mice displayed significantly lower fungal burden and no weight loss compared to control mice, suggesting *C. albicans* targets EGFR to promote infection. However, cytokine levels were largely similar in both EGFR cKO and control mice, suggesting a minimal role for EGFR in K14+ tissues in immune activation during OPC. Given that EGFR is required for the induced endocytosis of hyphae during OPC (16), EGFR deficiency appears to protect against *C. albicans* infection by preventing the initial steps of fungal invasion. In a previous study, we demonstrated that *C. albicans* (via candidalysin) specifically phosphorylates EGFR without phosphorylating other ErbB family members (25). Nevertheless, in the absence of EGFR, it remains possible that other ErbB receptors are phosphorylated as a compensatory mechanism, and further studies are warranted to identify which signaling pathways contribute to cytokine induction in this context.

Our findings align with previous studies showing that pharmacological inhibition of EGFR reduces fungal burden in murine OPC (16, 25, 55). Similarly, EGFR inhibition protects against mucormycosis, where mice pre-treated with the EGFR inhibitor gefitinib and infected with *Rhizopus delemar* displayed a significantly reduced fungal burden in the lungs and brain and prolonged survival compared to untreated mice (81). In contrast, EGFR is protective during *Aspergillus fumigatus* infection, where EGFR inhibition leads to increased fungal burden and accelerated mortality in a mouse model of invasive pulmonary aspergillosis (82). These contrasting outcomes highlight the context-dependent role of EGFR in fungal infection and immunity.

Ubiquitination is a central post-translational modification in eukaryotic cells, regulating an array of cellular functions, including proteasomal degradation, cell signaling, endocytosis and receptor trafficking, and cell death (43, 47). Beyond EGFR ubiquitination, our data reveal that *C. albicans* infection triggers widespread changes in host protein ubiquitination and alters the expression of several ubiquitin pathway-associated genes, both *in vitro* and *in vivo*. Notably, the de-ubiquitinase *USP36* was upregulated in an *ECE1*-driven manner. USP36 is known to stabilize c-Myc (83) and regulate protein phosphatase methylesterase-1, an inhibitor of the tumor suppressor protein phosphatase 2A, which negatively controls ERK and Akt signaling (84). Thus, increased *USP36* expression may indirectly promote ERK and Akt activation. In contrast, *USP13*, involved in the de-ubiquitination of the tumor suppressor p53 (85), was significantly downregulated, potentially leading to increased ubiquitination and degradation of p53. We also observed *ECE1*-dependent upregulation of *TNFAIP3* (A20), a ubiquitin-editing protein and a key negative regulator of inflammatory signaling. Together, these data suggest that *C. albicans* and Ece1p modulate both ubiquitination and de-ubiquitination processes to reshape host signaling during infection.

Manipulation of the host ubiquitin system by other pathogens has been documented (50, 51, 86). For example, the E6 protein secreted by the human papillomavirus promotes p53 degradation via ubiquitination (87); SARS-CoV-2 infection alters the host ubiquitinome in epithelial cells (88); and *Helicobacter pylori* suppresses USP7, reducing TRAF6 and p53 levels (89). These studies suggest that interference with the ubiquitin system may be a common mechanism across pathogens. A broader proteomics-based approach will be instrumental in identifying the specific host proteins and pathways targeted by ubiquitination during *C. albicans* infection.

While we cannot fully exclude the possibility that EGFR ubiquitination reflects a generalized epithelial stress response to candidalysin, several observations argue for a targeted mechanism. First, we observed that EGFR ubiquitination coincides with the recruitment of specific proteins (e.g., Grb2, AP2M1, and HRS), consistent with a regulated endocytic process rather than non-specific damage. Second, EGFR degradation appears to be lysosomal mediated rather than linked to loss of membrane integrity. The *Clostridium difficile* toxin B is also known to activate EGFR and the ERK-MAPK signaling pathway in human colonic epithelial cells (90). Conversely, melittin, the honeybee *Apis mellifera* venom peptide toxin, has been shown to inhibit MAPK signaling pathways in melanoma cells (91, 92). To our knowledge, toxin-induced EGFR ubiquitination and degradation is not widely reported, suggesting our findings are highly likely to be *C. albicans*-specific.

Together, our findings reveal a novel role for EGFR ubiquitination and trafficking in host–pathogen interactions during *C. albicans* infection. By actively targeting EGFR for degradation, the fungus interferes with epithelial signaling and invasion control, while the host attempts to compensate through transcriptional upregulation. These data underscore EGFR as a dynamic regulator of fungal pathogenesis that may be exploitable for therapeutic intervention, depending on the pathogen-specific context. Additionally, our results highlight ubiquitination as a key regulatory mechanism in the host response to *C. albicans*, with fungal modulation of de-ubiquitinases and ubiquitin-related pathways potentially shaping infection outcomes.

## MATERIALS AND METHODS

### Cell culture

Experiments were performed using the TR146 human buccal epithelial squamous cell carcinoma cell line (ECACC 10032305) (93). Cells were cultured in Dulbecco’s Modified Eagle Medium (DMEM) (DMEM/F-12, Gibco) supplemented with 15% fetal bovine serum (FBS) (Sigma-Aldrich) and 1% penicillin/streptomycin (Sigma-Aldrich) at 37°C with 5% CO_2_.

### *C. albicans* strains used for *in vitro* assays

Table 1 lists the *C. albicans* strains used in the *in vitro* assays. Strains were grown in yeast peptone dextrose (YPD) overnight (30°C, 180 rpm) and used the following day at a multiplicity of infection (MOI) between 1 and 10, as specified. In all experiments, MOI refers to the ratio of *C. albicans* cells to TR146 epithelial cells, set at 10:1, 5:1, or 1:1.

**TABLE 1:** Overview of *C. albicans* strains used for *in vitro* assays

*C. albicans* strain	Description and reference	Genotype
SC5314	Wild-type strain (94)	Blood isolate
BWP17 + CIp30	Derived from SC5314 (95)	*ura3::λimm434/ura3::λimm434 iro1::λimm434/iro1::λimm434 his1::hisG/his1::hisG arg4::hisG/arg4::hisG RPS1/rps1::(URA3-HIS1-ARG4)*
*ece1*Δ/Δ	Homozygous *ECE1* null mutant (22)	*ura3::λimm434/ura3::λimm434 iro1::λimm434/iro1::λimm434 his1::hisG/his1::hisG arg4::hisG/arg4::hisG ece1::HIS1/ece1::ARG4 RPS1/rps1::URA3*
*ece1*Δ/Δ + *ECE1*	*ECE1* re-integrant, homozygous *ECE1* null mutant complemented with a single copy of *ECE1* (22)	*ura3::λimm434/ura3::λimm434 iro1::λimm434/iro1::λimm434 his1::hisG/his1::hisG arg4::hisG/arg4::hisG ece1::HIS1/ece1::ARG4 RPS1/rps1::(URA3-ECE1)*
*ece1*Δ/Δ + *ECE1*_Δ184–279_	Candidalysin null mutant, homozygous *ECE1* null mutant complemented with a single copy of *ECE1* with the candidalysin encoding region deleted (22)	*ura3::λimm434/ura3::λimm434 iro1::λimm434/iro1::λimm434 his1::hisG/his1::hisG arg4::hisG/arg4::hisG ece1::HIS1/ece1::ARG4 RPS1/rps1::(URA3-ECE1Δ184-279)*
*als3*Δ/Δ (M1284)	Homozygous *ALS3* null mutant (96)	*als3::ARG4/als3::HIS1 ura3::λimm434/ura3::(URA3-IRO1)*

### *C. albicans* strains used for *in vivo* experiments

For *in vivo* studies, we used *C. albicans* WT AHY940, which is an isogenic strain to SC5314, except it is *LEU2* heterozygous to facilitate the integration and recycling of the CRISPR cassette. This allows for markerless gene editing, which is a superior method of mutant strain construction compared to previous techniques (as described previously [74]). The *ece1*Δ/Δ and *als3*Δ/Δ strains were constructed using CRISPR mutagenesis in the AHY940 background, which have no strain autotrophies and therefore were the mutants of choice for *in vivo* experiments. Strains were grown overnight (30°C, 180 rpm) and re-suspended in PBS the next day at a cell density of 1 × 10^7^ cells/mL.

### RNA isolation from *C. albicans* and RT-qPCR analysis

*C. albicans* strains were grown overnight in YPD medium at 30°C with shaking (200 rpm). Cells were harvested by centrifugation, washed twice in PBS, and used to prepare two diluted cultures per strain: in YPD and in Roswell Park Memorial Institute medium with 10% FBS. Cultures were incubated for 4 h at 30°C or 37°C, respectively. Cells were collected by centrifugation (2,500 × *g*, 3 min, 4°C) and flash-frozen in liquid nitrogen.

For RNA extraction, pellets were re-suspended in 400 µL AE buffer (50 mM sodium acetate, pH 5.2; 10 mM EDTA), mixed with 37.5 µL 25% SDS and 400 µL of phenol (pH 4.3), and incubated at 65°C for 20 min with intermittent vortexing. Samples were placed on ice for 5 min and centrifuged (17,000 × *g*, 15 min, 4°C), and the supernatant was collected, mixed via vortexing with 500 µL of 100% chloroform, and centrifuged (10,000 × *g*, 15 min, 4°C). The supernatant was collected and vortexed with 400 µL of chloroform and centrifuged (10,000 × *g*, 15 min, 4°C). The supernatant was removed (200 μL), and RNA was precipitated by adding 20 μL 3 M sodium acetate and 200 μL 100% isopropanol. Tubes were gently inverted and incubated at 4°C for 1 h. Samples were centrifuged (17,000 × *g*, 20 min, 4°C), and pellets were washed with 1 mL of 70% RNAse-free ethanol and re-centrifuged (17,000 × *g*, 20 min, 4°C). The supernatant was removed, and the pellets were air-dried before being re-suspended in 20–50 µL of RNAse-free water. RNA concentration was measured using a NanoDrop 2000 Spectrophotometer (Thermo Scientific, Waltham, MA).

cDNA was synthesized using the High-Capacity cDNA Reverse Transcriptase Kit (Applied Biosystems by Thermo Fisher Scientific, #4368814). RT-qPCR was performed with 100 ng cDNA, SYBR Green Master Mix (Qiagen, #208056), and 500 nM primers (Table 2) on a QuantStudio 7 Flex Real-Time PCR System. Gene expression was analyzed using the ΔΔCT method, with *ACT1* as the housekeeping gene and yeast-morphology wild-type *C. albicans* AHY940 as the reference control.

**TABLE 2:** Primers used to quantify gene expression in *C. albicans*

Gene^*a*^	Forward (F) or reverse (R)	Primer (5′−3′)
*ACT1*	F	ACTACCATGTTCCCAGGTATTG
*ACT1*	R	CCACCAATCCAGACAGAGTATT
*ECE1*	F	CTTTATCTTCTCAAGCTGC
*ECE1*	R	CAACAACAGAATCAATATCTTC

^*a*^ *ACT1*, actin; *ECE1*, extent of cell elongation 1.

### EGF

Human recombinant EGF (PeproTech) was reconstituted in water at a concentration of 83 µg/mL, and cells were stimulated with 100 ng/mL EGF for the indicated time.

### RNA isolation from TR146 cells and RNA-seq analysis

TR146 cells were infected with *C. albicans* strains or PBS for 4 h at an MOI of 10 or treated with 15 µM candidalysin (a concentration previously shown to elicit epithelial signaling without inducing cytotoxicity [22, 25, 70]) or water for 0.5, 2.0, and 6.0 h. Total RNA from TR146 cells was extracted using a Nucleospin II kit (Macherey-Nagel) following the manufacturer’s instructions and assessed for quality using a Bioanalyzer with an Agilent RNA 600 Nano Kit. RNA-seq libraries were prepared with a TruSeq RNA Sample Preparation Kit v.2 (Illumina) according to the manufacturer’s protocol and sequenced as paired-end reads. Raw sequencing data (FASTQ files) were aligned to the human reference genome (GRCh38, Ensemble release 92) using Kallisto (97) to obtain transcript abundance estimates. Transcript-level counts and transcripts per million (TPM) values were summarized to the gene level using the R package *tximport* (98). Genes with an Entrez ID and a mean TPM of >1 across samples were retained for analysis. Differential expression was performed with DESeq2, using Wald tests and Benjamini–Hochberg correction (FDR < 0.01). Log_2_ fold changes were used for heatmap visualization, and *padj* is reported in the text. The RNA-seq data set used in this study is associated with a currently (at the time of writing) unpublished data set from a preprint (99) (DOI: https://doi.org/10.21203/rs.3.rs-2159406/v1).

### Preparation of oral epithelial cell lysate

After treatment or infection, cells were lysed in ice-cold RIPA lysis buffer (50 mM Tris-HCl, pH 7.5, 150 mM NaCl, 1% Triton X-100, 1% sodium deoxycholate, 0.1% sodium dodecyl sulfate [SDS], and 20 mM EDTA) supplemented with phosphatase and protease inhibitors (1% vol/vol) (Thermo Scientific) and centrifuged at 13,300 × *g* for 10 min at 4°C. The Microplate Bicinchoninic Acid Protein Assay Kit (Thermo Scientific, #23252) was used for protein quantification. Samples were prepared by mixing 10 μg of protein lysate with Laemmli SDS sample buffer (4×) (J63615, Thermo Scientific), dithiothreitol (10×), and PBS.

### Immunoprecipitation of oral epithelial proteins

For immunoprecipitations, cells were lysed in ice-cold Pierce IP Lysis Buffer (25 mM Tris-HCl, pH 7.4, 150 mM NaCl, 1 mM EDTA, 1% NP-40, and 5% glycerol; Pierce, #87788) supplemented with protease and phosphatase inhibitors (1:100 dilution) (Sigma Aldrich) and DUB inhibitor PR-619 (Tocris Bioscience). Lysates were passed through a 25-gage needle five times and centrifuged at 13,300 × *g* for 10 min at 4°C. The lysates were pre-cleared (to minimize non-specific binding) by incubating with pre-washed Pierce Protein A/G Magnetic Beads (Thermo Scientific) for 1 h at 4°C on a rotating wheel. Beads were then separated from the cleared lysates with a magnetic stand, and the clarified lysates were kept for IP. Five hundred micrograms of cell lysate was combined with IP antibody (Table 3) and lysis buffer, and the mixture was incubated overnight on a rotating wheel at 4°C.

**TABLE 3:** Antibodies used for immunoprecipitation

Antibody^*a*^	Dilution	Company	Product code
EGF receptor mouse mAb (IP specific)	1:100	Cell Signaling Technology	2256S
Mouse mAb IgG2b isotype control	1:100	Cell Signaling Technology	53484S

^*a*^ EGF, epidermal growth factor; IP, immunoprecipitation; mAb, monoclonal antibody.

The following day, the lysate/IP antibody mixture was added to pre-washed A/G Magnetic Beads and incubated with rotation at 4°C for 1 h. The beads were then collected with a magnetic stand, and the supernatant was removed. The beads were washed three times with lysis buffer and once with water and boiled in SDS-PAGE buffer for 10 min at 95°C. Samples were electrophoresed on 4%–12% SDS-PAGE gradient gels prior to Western blotting.

### EGFR degradation assay with Bafilomycin A1 and MG-132

Cells were pre-treated with lysosomal inhibitor Bafilomycin A1 (0.25 μM) (Cell Signaling Technology, #54645S), proteasomal inhibitor MG-132 (10 μM) (Cell Signaling Technology, #2194S) or DMSO (control) for 1 h, then stimulated with PBS (control), 100 ng/mL EGF for 6 h or infected with SC5314 (MOI 5) for 10 h. Cell lysates were collected and separated by SDS-PAGE for Western blotting.

### Western blotting

Proteins were resolved by electrophoresis on gradient SDS-PAGE gradient gels (Bolt Bis-Tris Plus Mini Protein Gels, 4%–12%; Invitrogen, #NW04125BOX). Following electrophoresis, proteins were transferred onto nitrocellulose membranes (Bio-Rad). Membranes were blocked in 5% skimmed milk in Tris-buffered saline containing 0.1% Tween 20 (TBST) for 1 h with gentle shaking. After washing once with TBST, membranes were incubated with primary antibody (Table 4) with gentle agitation overnight at 4°C. The following day, membranes were washed three times for 5 min with TBST. Membranes were subsequently probed with secondary antibody (rabbit or mouse) or Clean Blot IP Detection Reagent (HRP) for IP samples (Table 5) for 1 h at room temperature and then washed six times for 5 min with TBST. Finally, the proteins were detected with the Immobilon Western Chemiluminescent HRP Substrate (Merck Millipore) and developed with an Odyssey Fc Imaging System (LI-COR). α-Actin was used as a loading control. Densitometry quantification was performed using Image Studio Lite (LI-COR Biosciences) software. For ubiquitination blots, densitometry was performed on the entire smear using identical area dimensions across samples.

**TABLE 4:** Primary antibodies used for Western blotting

Antibody^*a*^	Species	Dilution	Company	Product code
Total EGFR	Rabbit	1:1,000	Cell Signaling Technology	2232
pEGFR Y1045	Rabbit	1:1,000	Cell Signaling Technology	2237
Ubiquitin	Rabbit	1:1,000	Cell Signaling Technology	43124
HRS	Rabbit	1:1,000	Cell Signaling Technology	15087
GRB2	Rabbit	1:1,000	Cell Signaling Technology	3972
AP2M1	Rabbit	1:1,000	Cell Signaling Technology	68196
α-Actin	Mouse	1:10,000	Merck Millipore	MAB1501

^*a*^ AP2M1, adaptor related protein complex 2 subunit mu 1; EGFR, epidermal growth factor receptor; Grb2, growth factor receptor-bound protein 2; HRS, hepatocyte growth factor receptor tyrosine kinase substrate.

**TABLE 5:** Secondary antibodies used for Western blotting

Antibody^*a*^	Species	Dilution	Company	Product code
Peroxidase-conjugated AffiniPure goat anti-mouse	Goat	1:20,000	Jackson Immunoresearch	115-035-062
Peroxidase-conjugated AffiniPure goat anti-rabbit	Goat	1:10,000	Jackson Immunoresearch	111-035-003
Clean Blot IP detection reagent (HRP)	Not applicable	1:350	Thermo Fisher Scientific	21230

^*a*^ HRP, horseradish peroxidase; IP, immunoprecipitation.

### Animals

Animal work was performed according to institutional and UK Home Office guidelines. C57BL/6J, EGFR^fl/fl^ K14^Cre-^ (control), and EGFR^fl/fl^ K14^Cre+^ tissue-specific K14+ conditional EGFR knockout (EGFR cKO) mice (generated in-house) were housed in a dedicated facility at King’s College London in a 12 h light–dark cycle with access to water and standard rodent chow *ad libitum*.

### OPC

To induce EGFR cKO, tamoxifen (5 mg/kg) was administered to the mice via oral gavage for five consecutive days. Mice were then rested for 5 days. On day 0, mice were weighed and anesthetized with 75 mg/kg ketamine and 1 mg/kg Domitor and placed on heat pads. Lacri-Lube eye ointment was given to protect from eye dryness. To perform the infection, cotton swabs soaked in 1 × 10^7^ cells/mL *C. albicans* culture in PBS were placed sublingually for 75 min. Mice were injected with atipamezole to reverse the anesthesia. Mice were weighed daily. On day 2, mice were sacrificed, and tongues were excised and halved and used for quantification of fungal burden (CFU) and RNA extraction.

### Quantification of CFU in tongue tissue

Tubes containing half tongues were weighed to determine tissue quantity. Tongues were homogenized in 1 mL of DPBS using a gentleMACS Dissociator (Miltenyi Biotec). The tongue homogenate solution was aliquoted in a sterile microfuge tube. Three 10-fold serial dilutions (1:10, 1:100, and 1:1,000) were performed for each tongue homogenate sample. The neat sample and dilutions were plated in duplicate on YPD agar plates containing 50 µg/mL chloramphenicol and incubated for 24 h at 37°C. Colonies were counted, and CFU was calculated according to tissue weight.

### RNA isolation from tongue tissue

Tongue tissue was homogenized in 600 µL RLT buffer (RNeasy Plus Kit) and processed according to the manufacturer’s instructions. cDNA was synthesized using a High-Capacity cDNA Reverse Transcription Kit (Applied Biosystems by Thermo Fisher Scientific, #4368814) and used for qPCR to quantify gene expression of different genes using a QuantiNova SYBR Green PCR Kit (Qiagen). The primers used are presented in Table 6. Gene expression was normalized to the housekeeping gene GAPDH, and the relative gene expression to uninfected mice was calculated (ΔΔCt).

**TABLE 6:** Primers used to quantify gene expression in tongue tissue

Gene^*a*^	Forward (F) or reverse (R)	Primer (5′−3′)
*Gapdh*	F	CTAATGACCACAGTCCATTC
*Gapdh*	R	GATGGGATGATGTTTTGGTG
*Il17a*	F	CCCCTTTACACCTTCTTTTC
*Il17a*	R	ACGTTTCTCAGCAAACTTAC
*Cxcl1*	F	AAAGATGCTAAAAGGTGTC
*Cxcl1*	R	GTATAGTGTTGTCAGAAGCC
*Defb3*	F	ACCTTCTGTTTGCATTTCTC
*Defb3*	R	GGTCTTCTCTATTTTCTCTTGC
*Il6*	F	AAGAAATGATGGATGCTACC
*Il6*	R	GAGTTTCTGTATCTCTCTGAAG
*Ubc*	F	GAGACGATGCAGATCTTTG
*Ubc*	R	ATGTTGTAGTCTGACAGGG
*Egfr*	F	GAATTTCTAGTTCTCGTGGG
*Egfr*	R	CTGTCGCAAAGTTTGTAATG
*Tnfaip3*	F	CTGTCAGAAGTGTTTCATCG
*Tnfaip3*	R	CTTGATCTCAGCTGTTCTTC
*Usp36*	F	ACACATACGATCCCTACTTG
*Usp36*	R	CCTTCTTCTTACACTTAGCAC
*Usp13*	F	AGAGAGCGTATGTAGGAAAC
*Usp13*	R	ATTTGTGTGTTGAAGTCCTG

^*a*^ *Cxcl1*, C-X-C motif chemokine ligand 1;* Defb3*, defensin beta 3; *Egfr*, epidermal growth factor receptor; *Gapdh*, glyceraldehyde-3-phosphate dehydrogenase; *Il*, interleukin; *Tnfaip3*, tumor necrosis factor alpha-induced protein 3; *Ubc*, ubiquitin C;* Usp*, ubiquitin-specific protease.

### Protein extraction from tongue tissue

Tongue homogenate (see quantification of CFU) was pelleted (400 × *g*, 10 min, 4°C) and lysed in 250 μL of RIPA buffer supplemented with protease, phosphatase, and de-ubiquitinating enzyme inhibitors. Samples were prepared (50 μg in 50 μL) and separated by SDS-PAGE for Western blotting.

### Histology

To assess K14 and EGFR levels *in vivo*, EGFR cKO and control mice were sacrificed, and tongues were excised, formalin-fixed, and paraffin-embedded. Thin sections (6 μm) were cut, mounted onto slides, and prepared. Antigen retrieval was carried out in a pre-heated microwave pressure cooker for 5 min using citric acid buffer at pH 6.4. Sections were then incubated in blocking buffer (1% BSA in TBS and sodium azide) to reduce non-specific binding. After removing the blocking buffer, sections were incubated with primary antibodies overnight at room temperature. The following primary antibodies were used: EGFR (1:2,000; Abcam ab32077) and K14 (keratin 14) (1:5,000; BioLegend Polyclonal K14 #905304). Sections were washed with TBS and incubated with a biotinylated secondary antibody (1:200, Vectorlab BA-1000) for 1 h at room temperature. In parallel, streptavidin–HRP conjugate (StreptABC-HRP, Vector Labs) was prepared and allowed to conjugate for 30 min in TBS. Following another rinse and a 5 min wash in TBS, slides were incubated in StreptABC-HRP for 30 min at room temperature. After additional washes, sections were developed using DAB substrate for 10 min with gentle agitation. Slides were washed under running tap water for 5 min. For counterstaining, slides were incubated in hemalum for 2 min, washed until clear, and optionally differentiated in 0.5% HCl in 70% IMS, depending on nuclear staining intensity. Sections were then washed thoroughly in water, dehydrated in 100% IMS (4 × 2 min), and cleared in xylene (2 × 5 min). Finally, slides were mounted using DPX mountant, allowed to set at room temperature, and imaged using a ZEISS Axioscope.

### Statistical analysis

To analyze data and create graphs, GraphPad Prism 9 software (GraphPad Software Inc, La Jolla, CA) was used. A Shapiro–Wilk test was first used to determine data normality. If data were normal, a parametric test (ordinary one-way ANOVA with multiple comparison Dunnett test or unpaired *t*-test) was performed. If data were not normal, a non-parametric test (Kruskal–Wallis with multiple comparison Dunn’s test or Mann–Whitney test) was used. A *P* value of <0.05 was considered significant.

## SUPPLEMENTAL MATERIAL

Supplemental figures mbio.03448-25-s0001.pdf Figures S1-S4.

ASM does not own the copyrights to Supplemental Material that may be linked to, or accessed through, an article. The authors have granted ASM a non-exclusive, world-wide license to publish the Supplemental Material files. Please contact the corresponding author directly for reuse.
