# Supplementary material for: Candida albicans-induced ubiquitination of EGFR reveals novel host–fungal interaction pathways
Source: mBio. 2026 Jan 12;17(2):e03448-25. doi: 10.1128/mbio.03448-25 (PMC12892998; doi:10.1128/mbio.03448-25)
Supplement: Supplemental figures — Figures S1-S4. [file mbio.03448-25-s0001.pdf]

Supplemental Material

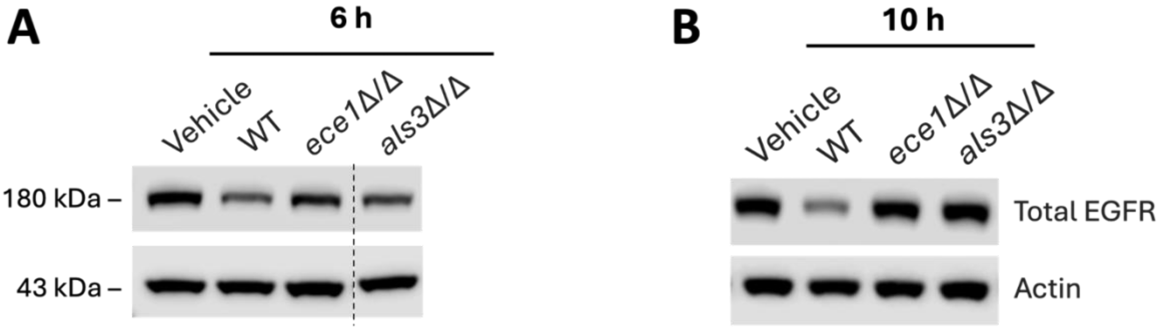

Supplemental Figure 1. *C. albicans* promotes EGFR degradation through Ece1p and Als3p. (A-B) TR146 cells were infected with different *C. albicans* strains (wild-type (WT) SC5314, *ece1*Δ/Δ and *als3*Δ/Δ) or stimulated with PBS as a vehicle control for 6 h (MOI 5) and 10 h (MOI 1). Cell lysates were electrophoresed on gradient gels to detect total EGFR and α-actin via western blot. Dashed vertical lines indicate omitted, extraneous portions of blot images. Data are representative of three independent experiments.

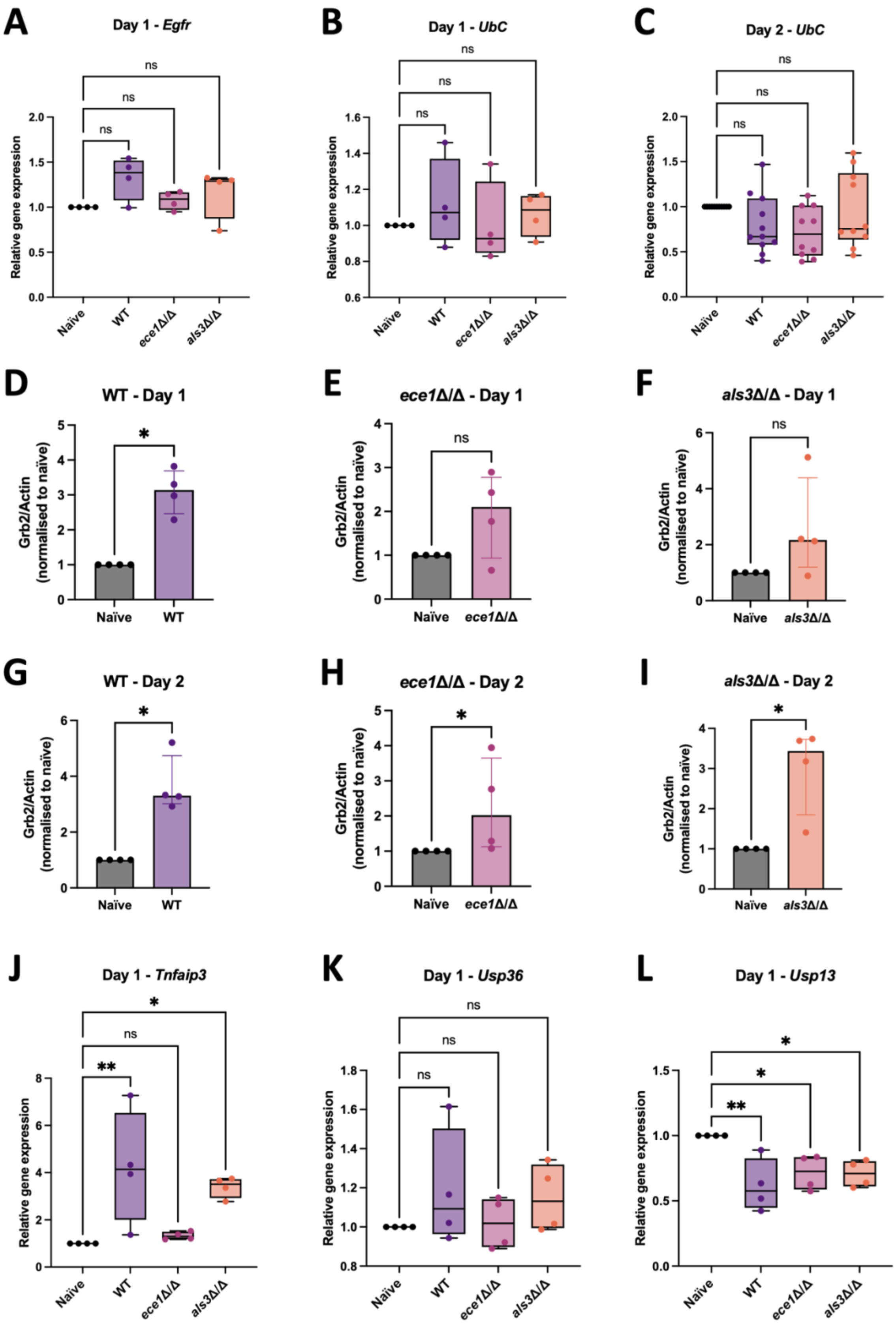

A
Day 1 - Egfr
Relative gene expression
Naïve
WT
ece1Δ/Δ
als3Δ/Δ
ns
B
Day 1 - UbC
C
Day 2 - UbC
D
WT - Day 1
Grb2/Actin (normalised to naïve)
E
ece1Δ/Δ - Day 1
F
als3Δ/Δ - Day 1
G
WT - Day 2
H
ece1Δ/Δ - Day 2
I
als3Δ/Δ - Day 2
J
Day 1 - Tnfaip3
K
Day 1 - Usp36
L
Day 1 - Usp13

Supplemental Figure 2. *C. albicans* dynamically regulates *Egfr*, ubiquitin pathway-associated genes and Grb2 in an oropharyngeal candidiasis mouse model. C57BL/6J mice were sublingually infected with wild-type (WT) *C. albicans* AHY940 and mutant strains *ece1*Δ/Δ and *als3*Δ/Δ, and tongues were harvested at 24 and 48 h post-infection. Gene expression of (**A**) *Egfr,* (**B-C**) *UbC*, (**J**) *Tnfaip3*, (**K**) *Usp36* and (**L**) *Usp13* was quantified using ΔΔCt relative to the naïve control. Data was collected from one to three independent experiments with 3-4 mice/group. Data are plotted as box plots, where the central line represents the median, the box indicates the interquartile range, and the whiskers extend to the minimum and maximum values. Individual data points represent biological replicates. A Shapiro-Wilk test was first used to determine data normality. If data were normal, a parametric test (ordinary one-way ANOVA with Dunnett's multiple comparison test) was performed. If data were not normal, a nonparametric test (Kruskal-Wallis test with Dunn's multiple comparison test) was used to determine statistical significance. (**D-I**) Relative protein levels of Figure 4B were quantified using densitometry. Target protein levels were normalised to actin and expressed relative to the naïve control, which was set to 1. Data are presented as median with interquartile range. Individual data points represent biological replicates. Statistical significance was determined using an unpaired non-parametric t-test (Kolmogorov-Smirnov test). ns, not significant, *$p < 0.05$, **$p < 0.01$.

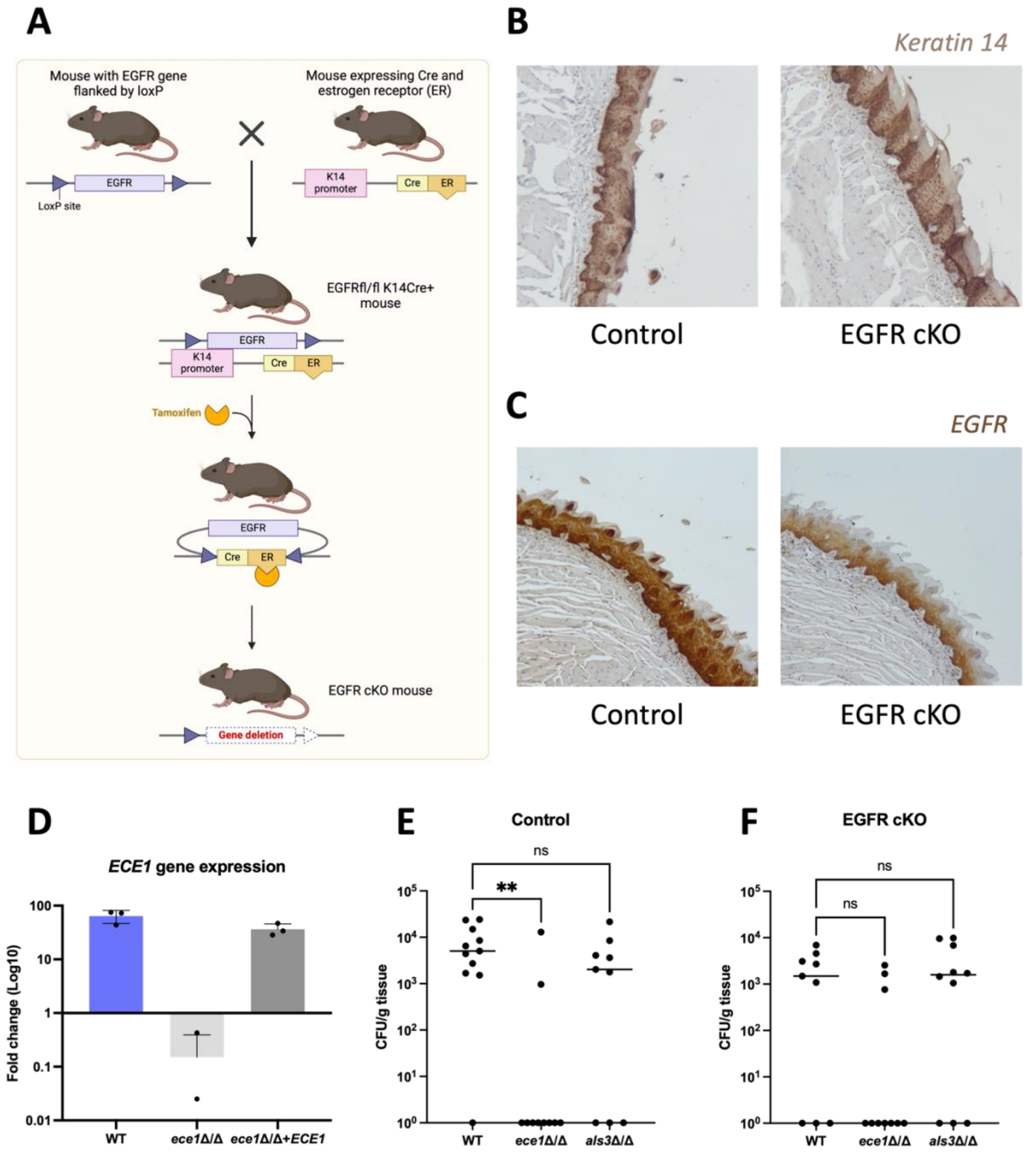

Supplemental Figure 3. Generation of EGFR cKO mice and infection with mutant strains *ece1*Δ/Δ and *als3*Δ/Δ. (**A**) K14-Cre/ERT transgenic mice were crossed with mice carrying an EGFR gene flanked by loxP sites, resulting in offspring capable of permitting tamoxifen-induced EGFR knockout in K14-expressing cells. Created with BioRender.com. Immunostaining of the epithelial layer of the tongue of control and EGFR cKO mice with (**B**) keratin 14 and (**C**) EGFR antibodies. (**D**) Wild-type (WT) *C. albicans* (AHY940), *ece1*Δ/Δ and *ece1*Δ/Δ+*ECE1* were cultured in hypha-inducing conditions for 4 h and *ECE1* gene expression was quantified. Data is presented as $\log_{10}$-fold change normalised to yeast-WT and is the mean and standard deviation of three biological repeats. (**E-F**) EGFR cKO

(EGFR$^{fl/fl}$ K14^{Cre+}) and control (EGFR$^{fl/fl}$ K14^{Cre-}) mice were sublingually infected with WT *C. albicans* AHY940 and mutant stains *ece1*Δ/Δ and *als3*Δ/Δ. Tongues were harvested on day 2 post-infection to measure colony-forming units (CFU). Data were transformed by Y=Y+K, K=1. CFU data were plotted on a log scale where the central line represents the median. Individual data points represent biological replicates. A Shapiro-Wilk test was first used to determine data normality. If data were normal, a parametric test (ordinary one-way ANOVA with Dunnett's multiple comparison test) was performed. If data were not normal, a nonparametric test (Kruskal-Wallis test with Dunn's multiple comparison test) was used to determine statistical significance. ns, not significant, $**p < 0.01$. Data was collected from three experiments with 3-4 mice/group.

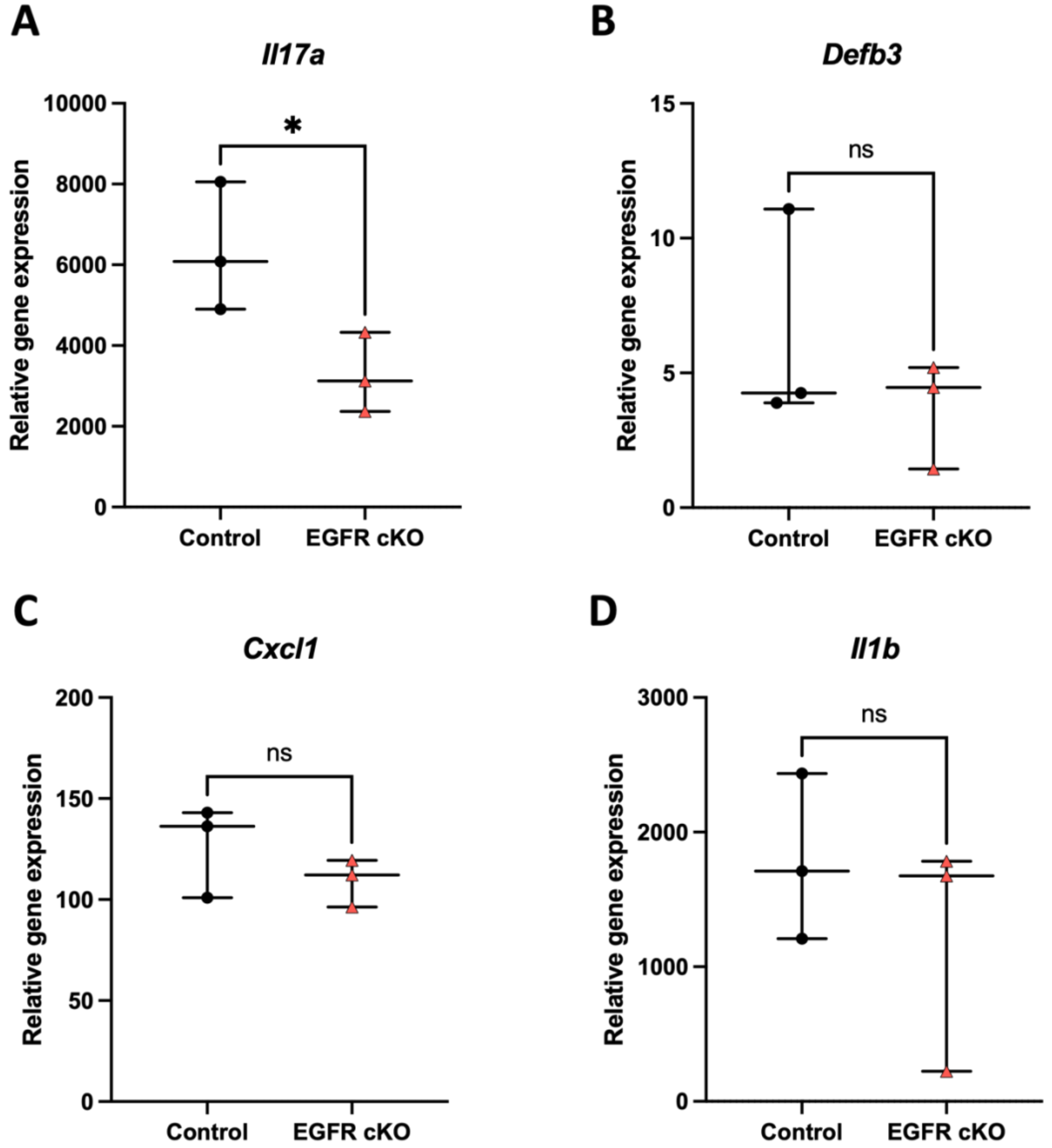

Supplemental Figure 4. Mucosal immunity to *C. albicans* infection is not significantly impaired in EGFR cKO mice at day 1. EGFR cKO (EGFR$^{fl/fl}$ K14^{Cre+}) and control (EGFR$^{fl/fl}$ K14^{Cre-}) mice were sublingually infected with wild-type (WT) *C. albicans* AHY940. Gene expression of (**A**) *Il17a*, (**B**) *Defb3*, (**C**) *Cxcl1* and (**D**) *Il1b* at day 1 was quantified using ΔΔCt relative to the naive control. Data are plotted as box plots, where the central line represents the median and the whiskers extend to the minimum and maximum values. Individual data points represent biological replicates. A Shapiro-Wilk test was first used to determine data normality. If data were normal, a parametric test (unpaired t-test) was performed. If data were not normal, a nonparametric test (Mann-Whitney test) was used to determine statistical significance. Data was collected from one independent experiment with 3 mice/group. ns, not significant, *$p < 0.05$.
